# Identification of lysosomotropism using explainable machine learning and morphological profiling cell painting data†

**DOI:** 10.1039/d4md00107a

**Published:** 2024-05-24

**Authors:** Aishvarya Tandon, Anna Santura, Herbert Waldmann, Axel Pahl, Paul Czodrowski

**Affiliations:** a Department of Chemical Biology, Max-Planck-Institute of Molecular Physiology Otto-Hahn-Str. 11 Dortmund Germany herbert.waldmann@mpi-dortmund.mpg.de axel.pahl@mpi-dortmund.mpg.de; b Department of Chemistry, Johannes Gutenberg University Mainz Mainz Germany czodpaul@uni-mainz.de

## Abstract

Lysosomotropism is a phenomenon of diverse pharmaceutical interests because it is a property of compounds with diverse chemical structures and primary targets. While it is primarily reported to be caused by compounds having suitable lipophilicity and basicity values, not all compounds that fulfill such criteria are in fact lysosomotropic. Here, we use morphological profiling by means of the cell painting assay (CPA) as a reliable surrogate to identify lysosomotropism. We noticed that only 35% of the compound subset with matching physicochemical properties show the lysosomotropic phenotype. Based on a matched molecular pair analysis (MMPA), no key substructures driving lysosomotropism could be identified. However, using explainable machine learning (XML), we were able to highlight that higher lipophilicity, basicity, molecular weight, and lower topological polar surface area are among the important properties that induce lysosomotropism in the compounds of this subset.

## Abstract 2

Explainable ML was used to identify important chemical structural properties that contribute to lysosomotropism.

## Graphical abstract

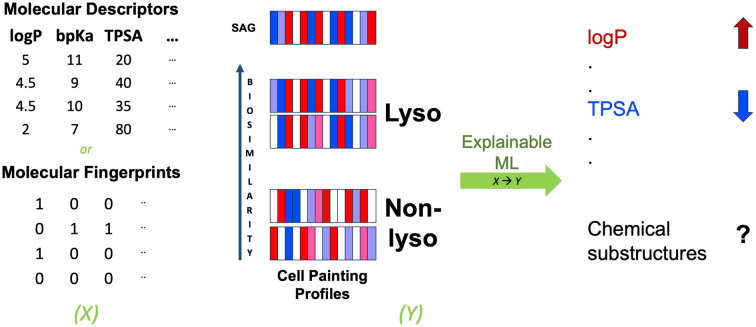

## 1 Introduction

**Fig. 1:**
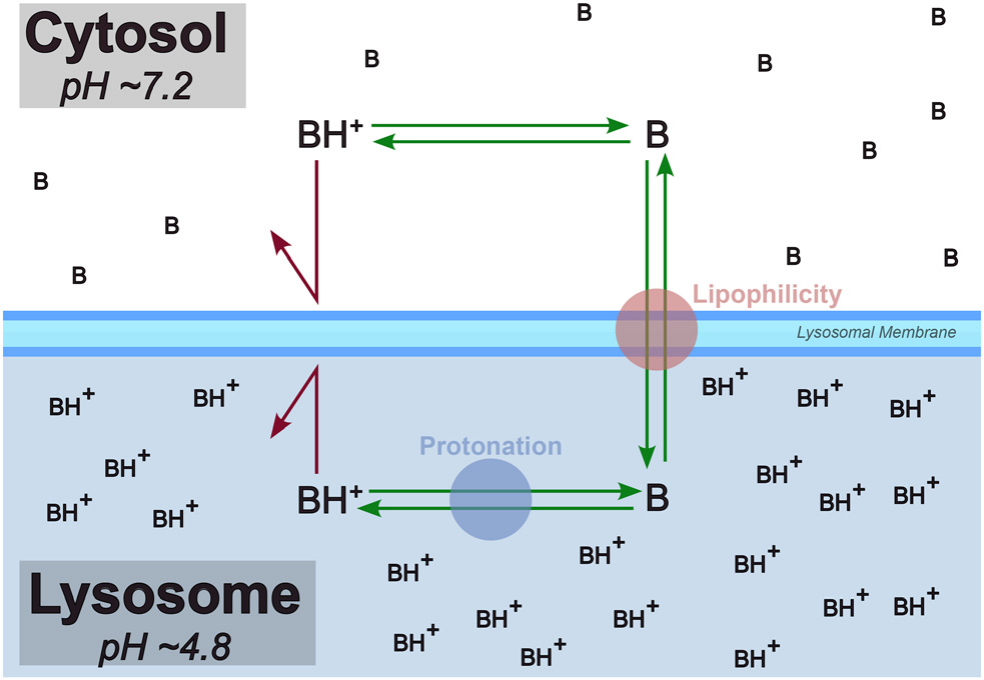
Lysosomotropism is primarily believed to be driven by lipophilicity and protonation state of the compound. In this diagram, “B” is a lysosomotropic compound and “BH^+^” is its protonated state. Illustration inspired by Kuzu *et al.* (2017).^4^

The lysosome plays a crucial role in the cellular degradation of biopolymers and in processes, such as apoptosis, autophagy, and cell signaling. Lipophilic small molecules and drugs that carry a basic moiety can accumulate in the lysosome, by passing the lysosomal membrane in their neutral form, getting trapped in the compartment due to protonation at the lower pH (Fig. 1). The pathophysiological consequences of this phenomenon termed lysosomotropism are not yet fully understood, but impairment of lysosomal functionality can be linked to phospholipidosis and disturbed cholesterol homeostasis.^1–3^

Recently lysosomotropism has especially drawn attention in multiple drug repurposing studies targeting severe acute respiratory syndrome coronavirus 2 (SARS-CoV-2). This stems from the crucial involvement of cathepsin L, a lysosomal protease, in cleaving the SARS-CoV-2 spike protein and facilitating virus entry into host cells. By the accumulation of lysosomotropic drugs in the lysosome, the compartment's pH rises, rendering proteolytic enzymes inactive and impeding viral replication.^5–7^

Consequently, well-established lysosomotropic drugs, *e.g.* chloroquine and hydroxychloroquine, were initially promising drug repurposing candidates against SARS-CoV-2. However, these drugs failed to demonstrate significant clinical benefits.^6,8–13^

In a more general sense, lysosomotropism is also a phenomenon of various drugs. It occurs for structurally different compound classes, modes of actions and targets, and is independent of species and cell-type.^3,14^ For instance, lysosomotropic properties have been observed in anticancer compounds (tamoxifen, doxorubicin, daunorubicin, mitoxantrone), tyrosine kinase inhibitors (imatinib, dasatinib, sunitinib, sorefenib), β-blockers (propranolol), antihistamines (promethazine, astemizole, dimebon, desloratadine), and selective serotonin reuptake inhibitors (sertraline, paroxetine, fluoxetine, fluvoxamine).^4,15–17^

Given such broad range of interests, several groups have contributed towards the measurement, quantification and prediction of lysosomotropism, see *e.g.* Nadanaciva *et al.* (2011), Ufuk *et al.* (2017), Schmitt *et al.* (2018) and Norinder *et al.* (2019).^15,18–20^ Highlighting the importance of identifying lysosomotropism early in the development process, Hu *et al.* (2023) recently developed and published models on phospholipidosis, a process related to lysosomotropism, using compound literature data. They also validated their results using a live-cell imaging assay.^21^

Compounds with a calculated log *P* (clog *P*) value greater than 2 and a basic p*K*_a_ (bp*K*_a_) value between 6.5 and 11, representing lipophilicity and basicity, respectively, are likely to be lysosomotropic.^15^ Here, we will refer to this cross-section of properties as the physicochemical window (“PhysChem window”). While lysosomotropism at first seems to be primarily driven by lipophilicity and protonation state of compounds, it has been established that not all molecules which have the suitable physicochemical properties are in fact lysosomotropic.^15,22^

The cell painting assay (CPA) is an unbiased, image-based phenotypic assay where morphological profiles consisting of hundreds of features are generated from the images of compound-treated and control cells.^23^ One typical use case of the CPA is the formation of target hypotheses for test compounds with unknown biological activity. Here profiles of test compounds are compared to those of reference compounds with annotated targets or pathways. However, many compounds with lysosomotropic properties induce a distinct phenotype in the CPA which is independent of their target activity.^3^ We have observed that comparing morphological profiles of test compounds to that of a known lysosomotropic agent – smoothened agonist (SAG), is a reliable surrogate for determining lysosomotropism.

Machine learning (ML) methods have become an integral part of drug discovery. Some prominent methods are QSAR, prediction of chemical reactions and retrosynthesis, and the generation of novel chemical structures.^24,25^ Programming packages such as LIME and SHAP offer “explainability” of a model, enabling the interpretation of its predictions.^26,27^ The transparency about a model's predictions inspires confidence in researchers to trust them, which is why these packages are gaining popularity and application in drug discovery.^28–30^ Similarly, input features found important by a bioactivity prediction model can be determined using such packages, and this information can be used to develop a hypothesis of the underlying mechanism of the bioactivity.

Herein, we investigate the lysosomotropism observed by the CPA using matched molecular pair analysis (MMPA) and explainable machine learning (XML) to understand which physicochemical descriptor and/or chemical substructures affect lysosomotropism in compounds with feasible basicity and lipophilic values.

With the MMPA, we aim to identify key substructures which are responsible for transformation of a lysosomotropic compound to a non-lysosomotropic compound, and *vice versa*. Similarly, by interpreting tree-based machine learning (ML) models with molecular fingerprints we addressed the identification of important substructures whose presence affects lysosomotropism. Finally, by interpreting ML models with molecular descriptors as input, the determination of physicochemical parameters which affect lysosomotropism is attempted.

## 2 Results

### 2.1 Determination of lysosomotropism

The cell painting assay (CPA) is a morphological profiling assay where six dyes are used to selectively stain different cell organelles and compartments, followed by high-content imaging and analysis, generating morphological fingerprints with hundreds of features.^23,31^ CPA is an unbiased assay, that can identify biological activity without requiring a prior target hypothesis, and is therefore particularly well-suited for the screening of new chemical entities of unknown activity. Comparison of the CP profile of a hit molecule with the profiles of reference compounds whose modes of actions and targets are known can then provide target hypotheses, potentially enabling target identification.^32–37^

In our implementation of post-imaging analysis following the feature calculation by the open-source software *CellProfiler*, 579 *Z*-scores of morphological features are deduced per compound. The *Z*-score of a morphological feature represents the difference between a morphological feature and its relative DMSO control. A compound's morphological profile (or simply its CP profile) is thereby a list of its *Z*-scores.^34^

Our processed CP data represents a total of 13 450 compounds. In this data, 3114 are reference compounds, whose biological activities are annotated, and 10 336 are internal research compounds. The internal research compounds primarily consist of natural products-inspired compounds and pseudo-natural products.^38,39^ 2065 compounds are present in the PhysChem window, and thereby are relevant to this study.

Induction, a measure of bioactivity, is the percentage of significantly altered features. Compounds with the induction value greater than or equal to 5 are considered bioactive.^34^ In the PhysChem window, 1196 compounds are bioactive, while 869 are not.

The CPA is a routine in-house screening assay at the Compound Management and Screening Center, Dortmund, and is used in identifying numerous biological clusters and pathways, among them lysosomotropism. The similarities between CP profiles can be measured by Pearson's similarity. The CP profiles are considered similar if their Pearson's similarity values are greater than 75%. Schneidewind *et al.* (2021) identified a biocluster in the CPA data whose mode of action is likely due to disturbed cholesterol homeostasis caused by lysosomotropism.^3^

Smoothened agonist (SAG), a well-established lysosomotropic compound, is present as a reference compound in the dataset and can be found in the reported biocluster. Because of its pronounced profile in the CPA, SAG was used as the reference compound for defining the lysosomotropic phenotype. This similarity score, termed as the lyso score, ranges from 0 (indicating no biosimilarity) to 100 (indicating full biosimilarity). Compounds with a lyso score above or equal to 75 were annotated as lysosomotropic given the high biosimilarity of their profile to the profile determined for SAG, the rest were labelled as non-lysosomotropic. Out of the 2065 compounds present in the PhysChem window, 1327 were labelled as non-lysosomotropic and the remaining 738 as lysosomotropic.

**Fig. 2:**
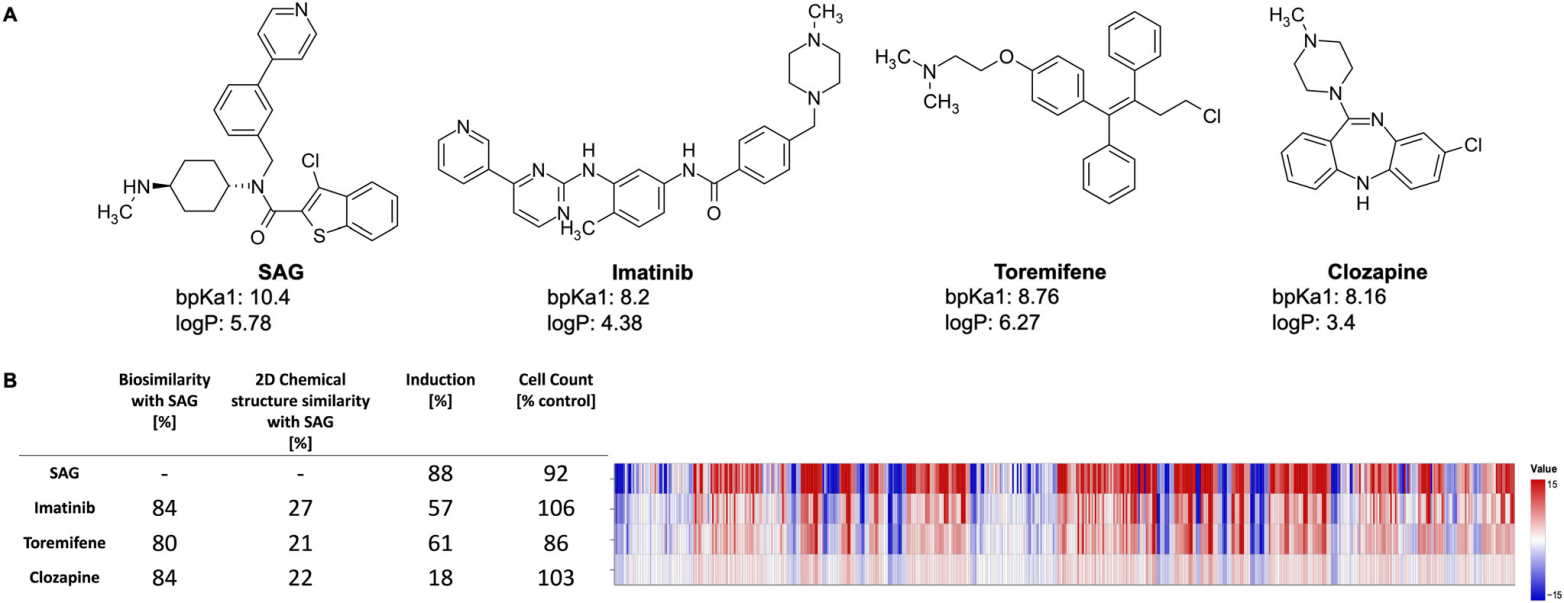
A) Different chemical structures of SAG, imatinib, toremifene, and clozapine with their calculated bp*K*_a_ and log *P* values. B) The CP profiles (579 features) of SAG, imatinib, toremifene, and clozapine shown as heatmaps. The values of each feature are normalized to the DMSO control. The blue color means that the value is decreased whereas the red color means that the value has increased. Despite different chemical structures and known pharmaceutical properties, the investigated compounds have similar morphological profiles.

Fig. 2 exemplarily shows three lysosomotropic reference compounds – imatinib, toremifene, and clozapine – and their CP profiles in comparison to SAG. The morphological profiles of these three compounds and that of SAG are very similar, although these compounds have different primary targets and chemical structures. Imatinib, a tyrosine kinase inhibitor, is primarily used to treat chronic myeloid leukemia,^40^ whereas toremifene, a selective non-steroidal estrogen receptor modulator, is administered to treat breast cancer.^41^ Clozapine is an anti-psychotic drug used in the treatment of severely ill patients with schizophrenia. While clozapine's mode of action is unknown, it is proposed to be an antagonist of dopamine and serotonin receptors.^42^

### 2.2 Matched molecular pair analysis (MMPA)

#### Concept and nomenclature

A MMP is formed by two compounds that differ from each other by a defined change at one or more specified positions.^43–45^ The point where the change takes place is referred to as the attachment point; the local environment of an attachment point is denoted as the context, or simply the environment. The part of the molecules that is identical is called constant, and the part that changes is named the variable. Consequently, the compounds belonging to a MMP can be converted to one another by the molecular transformation of variable A to variable B, *i.e.* A → B. Transformation types include, for instance, additions (H → X) or functional group replacements (*e.g.*, Cl → OMe) or linker/scaffold exchange. Every transformation is associated with a relative change in a property value in general (Δ*P*),^44^ here a change in the lyso score. Subsequently, the same transformations are grouped together and the statistics of the property changes are calculated (frequency of occurrence, Δ*P* distribution, average Δ*P*, *etc.*), which collectively yields the rules.^46,47^ Taken together, examination of analogous compounds can determine the contribution of each substituent or structural element to the overall property of the compound (assumption of additivity).^48^

Furthermore, it is anticipated that the effect of a substituent on the respective physicochemical/biological property can be generalized, *i.e.* that its contribution is transferable across compound series.^48^

Given that the method captures the implicit knowledge contained in the chemical dataset in a systematic and automated manner, the emergence of the rules is fully explainable (as it is easy to trace back to the underlying compound pairs), resembles the intuitive way of a chemist's thinking, and lacks the “black box” character, which is frequently raised as a point of critique concerning machine learning or other *in silico* methods utilized for (Q)SAR analysis.^45,46^

The MMP concept has been widely employed.^43–51^ However, to the best of our knowledge, the application of this approach to a CP data set and with a view to lysosomotropism has not been reported to date.

#### MMPA results

In total, 6220 MMPs were identified within the dataset of 2065 compounds described above. As a result of the significantly higher number of non-lysosomotropic compared to lysosomotropic compounds in the data set, many more MMPs are found in which both compounds are non-lysosomotropic, compared to the other two cases. For 956 MMPs the lysosomotropic property is altered upon the respective transformation, *i.e.* the lyso score threshold of 75 is passed.

Altogether, 4441 unique transformations have been found regardless of the lysosomotropism classification. However, >99% of them occur less than 7 times (Fig. S1) (ESI†). Similar numbers, as well as the Zipfian-shaped distribution of counts (number of occurrence), have already been described by Hussain and Rea (2010),^51^ among others.

**Fig. 3:**
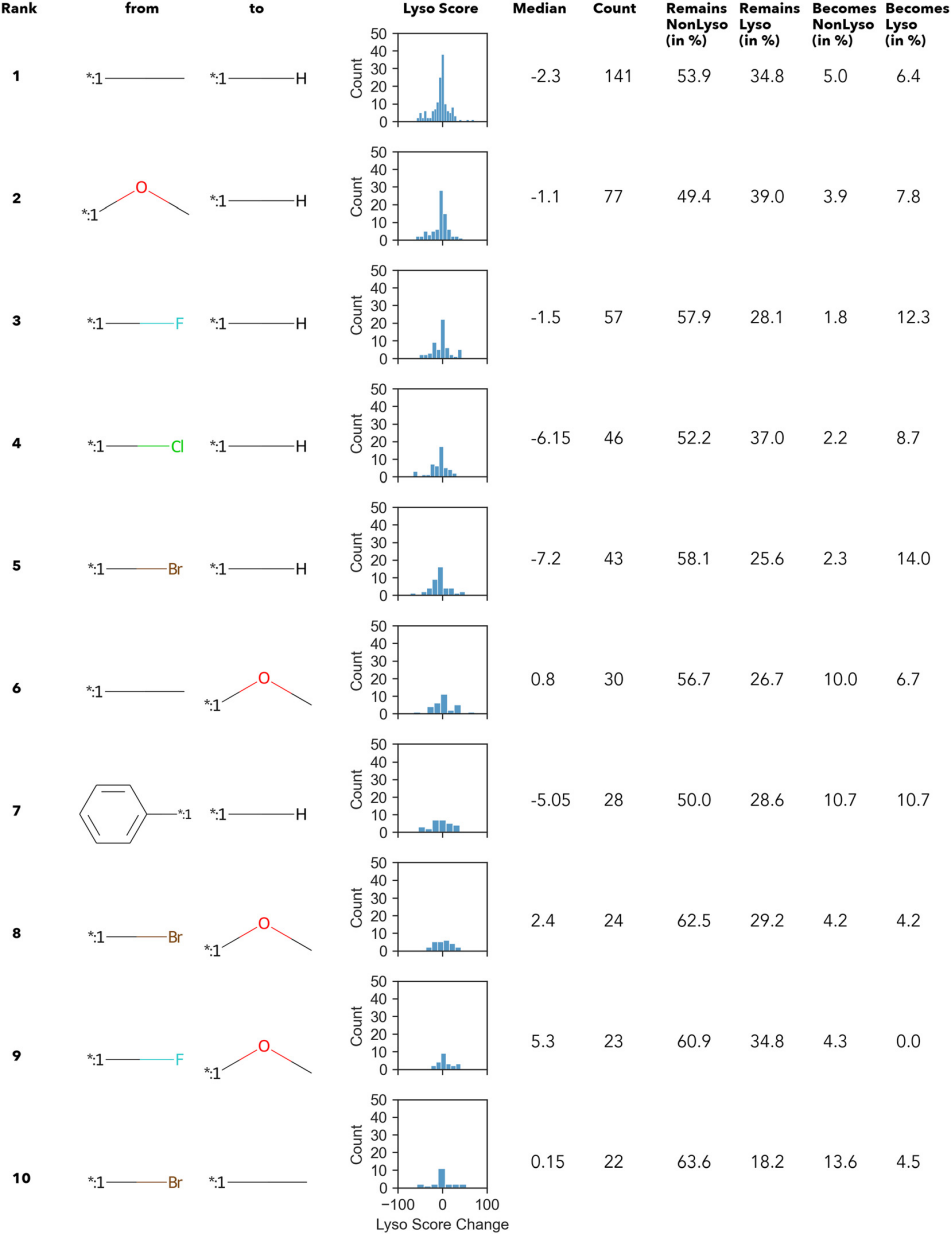
Top 10 transformations with respect to the count. Histograms display the distribution of the change in the lyso score value upon the respective transformation; additionally, the median value is given.

In summary, only 27 transformations occur ten times or more – of which the top 10 transformations with the highest numbers are shown in Fig. 3. Unsurprisingly, most of the “high-count” transformations found either resemble “simple” terminal group substitutions, where only a single atom is replaced, or functional group substitutions.

The Δ value is calculated by subtracting the lyso score of the “from” compound from that of “to” compound. In other words, if the Δ lyso score distribution is shifted to the right, the transformation is accompanied by an increase in lysosomotropism. However, in none of the “high-count” conversions shown in Fig. 3, the change in lyso score incidental to the transformations is in one direction only. The MMPA performed in this work intimates that there does not appear to be a dominant structural feature in the compound library under investigation that determines lysosomotropism.

### 2.3 Explainable machine learning

#### Molecular fingerprints

We chose 3 major categories of molecular fingerprints, Morgan fingerprints (the term is used interchangeably with ECFP here), MACCS keys, and Avalon fingerprints. All fingerprints mentioned here are binary in nature, and are generated by RDKit. Morgan fingerprints use a hashing function to map substructures of different radius to an index of a vector, and that vector can be folded to different sizes.^52,53^ We provided 3 different radii of 2, 3 and 4, thereby generated 3 different sets of these fingerprints for our data. MACCS keys encode absence or presence of 166 pre-defined structural features. Avalon fingerprints enumerate certain paths and feature classes of a molecular graph.^54^

#### Molecular descriptors

A molecular descriptor, as defined by R. Todeschini and V. Consonni, is “*the final result of a logic and mathematical procedure which transforms chemical information encoded within a symbolic representation of a molecule into an useful number*”.^55^

We used RDKit to generate one- and two-dimensional molecular descriptors of the compounds.^56^ Around 200 molecular descriptors can be generated by the RDKit. However, since we were aiming for explainability, we manually selected the descriptors which are intuitive, such as *NumHAcceptors*, *TPSA*, *FractionCSP3*, *fr_nitro*, *etc.* Some examples of unintuitive descriptors, which were removed, are *PEOE_VSA7*, *VSA_EState8*, *SMR_VSA6*, *etc.*

In total, 107 intuitive molecular descriptors were selected. These select descriptors are listed in the Table S1.† log *P* and bp*K*_a_ calculated by ChemAxon *cxcalc*, were also used additionally for one of the models.

#### Modelling with XGBoost

Decision tree models are basic tree-structured ML models. They can be easily understood and interpreted since their logical chain of arriving at a decision can be visualized. However, they suffer from drawbacks, such as overfitting and high computation cost.^57^ Gradient boosting is a technique which ensembles numerous weak models (here, decision trees) to make a prediction. This ensemble of trees usually improves both prediction performance and lowers computation cost, however the comprehensibility, which a single decision tree offers, is sacrificed. Extreme gradient boosting (XGBoost) is a ML algorithm (and the software library of the same name) which uses optimized gradient boosting, and is engineered to be highly efficient across different platforms and high dimension data.^58^

We prepared XGBoost binary classifiers as described below. Except for the *scale_pos_weight* hyperparameter, which was used to provide the weights of “Non-Lysosomotropic” and “Lysosomotropic” classes to control the class imbalance, default hyperparameters were used for training. The performances of the models trained with the default hyperparameters and with the optimized hyperparameters by the package Optuna^59^ were found similar, and thereby the default hyperparameters were used. All the models were trained on the internal data. Stratified 5-fold cross-validation with the balanced accuracy and the Cohen's kappa score as model performance metrics, was used to validate the models. The libraries present in the *Scikit learn* package were used for these calculations.^60^

#### Use of SHAP for feature importance

Shapley additive explanations (SHAP) is a model-agnostic, game-theory based approach to explain ML models. SHAP focuses on local explanations, *i.e.*, the impact of every input feature on the output of a single sample. This impact, a quantitative contribution of the feature, is called Shapley value (term used interchangeably with SHAP value) and is measured in log-odds.^27,61^ SHAP is increasingly used for model explanation in cheminformatics.^28,62^ We used the *TreeExplainer* from the SHAP Python package, since our models are tree-based. *TreeExplainer* has an important advantage over the default *kernel SHAP*. It computes exact Shapley values by taking advantage of the internal structure of the tree-based models with nominal computation power, whereas the default *kernel SHAP* uses an approximation of Shapley values to save the computation power it would need otherwise to calculate the exact values. Computing exact Shapley values allows global interpretation of the model by combining the local explanations.^63^

Due to the numerical nature of the descriptors, *TreeExplainer* could be used directly on the models trained on the molecular descriptors and various SHAP plots can be employed to study the descriptors and their importance on a data set. However, molecular fingerprints are binary in nature and especially in the case of Morgan fingerprints, multiple substructures can be encoded in the same bit. Thus, highlighting bits as important is unintuitive unless the substructures they encode are known. We used *X-FP*, a Python library, to compute the substructures of the bits which Morgan fingerprints encodes. We then used *X-FP*'s functionality to calculate feature importance by SHAP *TreeExplainer* and visualized these important bits and the substructures they encoded.^64^

#### Model training results

**Fig. 4:**
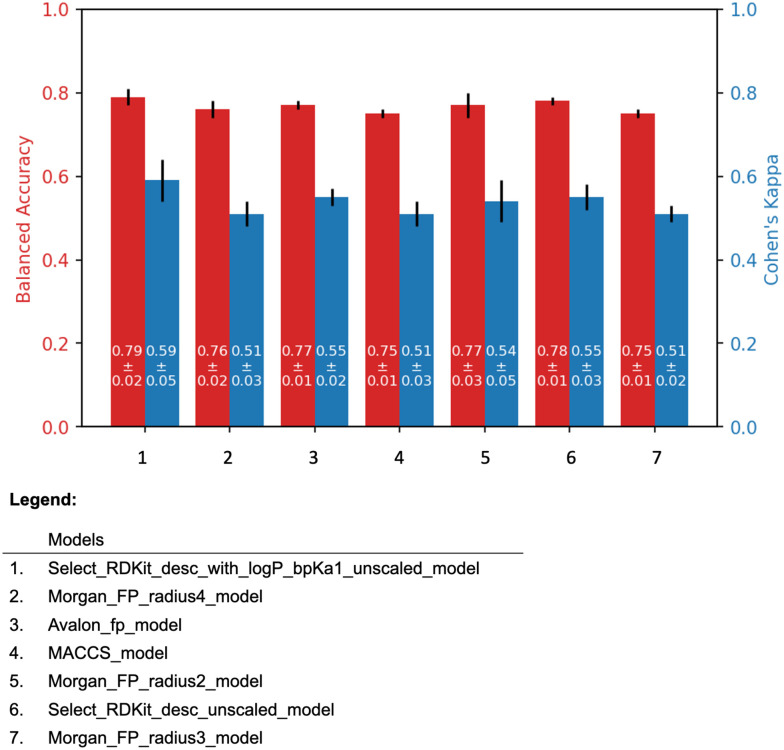
The 5-fold cross validation results of the all models. The black line on top of the bars indicates the standard deviation.

The 5-fold cross validation results of all the models are shown in Fig. 4. Post cross-validation, models were trained on the entire data, and validated on two different additional datasets.

The molecular descriptor model, “Select_RDKit_desc_with_logP_bpKa1_unscaled model”, and the molecular fingerprint model, “Morgan_FP_radius2_model” were selected as the representatives of their respective model types. For convenience, these models are referred to as the “Descriptor model” and the “Fingerprint model”, respectively.

The descriptor model has the average balanced accuracy of 0.79 with the standard deviation of 0.02, and the average Cohen's kappa score of 0.59 with the standard deviation of 0.05. Similarly, the fingerprint model has the average balanced accuracy of 0.77 with the standard deviation of 0.03, and the average Cohen's kappa score of 0.54 with the standard deviation of 0.05.

#### Time-split validation

After the model was developed and validated, new CPA measurements were performed. This allowed us to perform a time-split validation. This dataset consists a total of 156 compounds relevant to this study (located within the PhysChem window), where 114 are labelled as lysosomotropic and the remaining 42 as non-lysosomotropic.

**Fig. 5:**
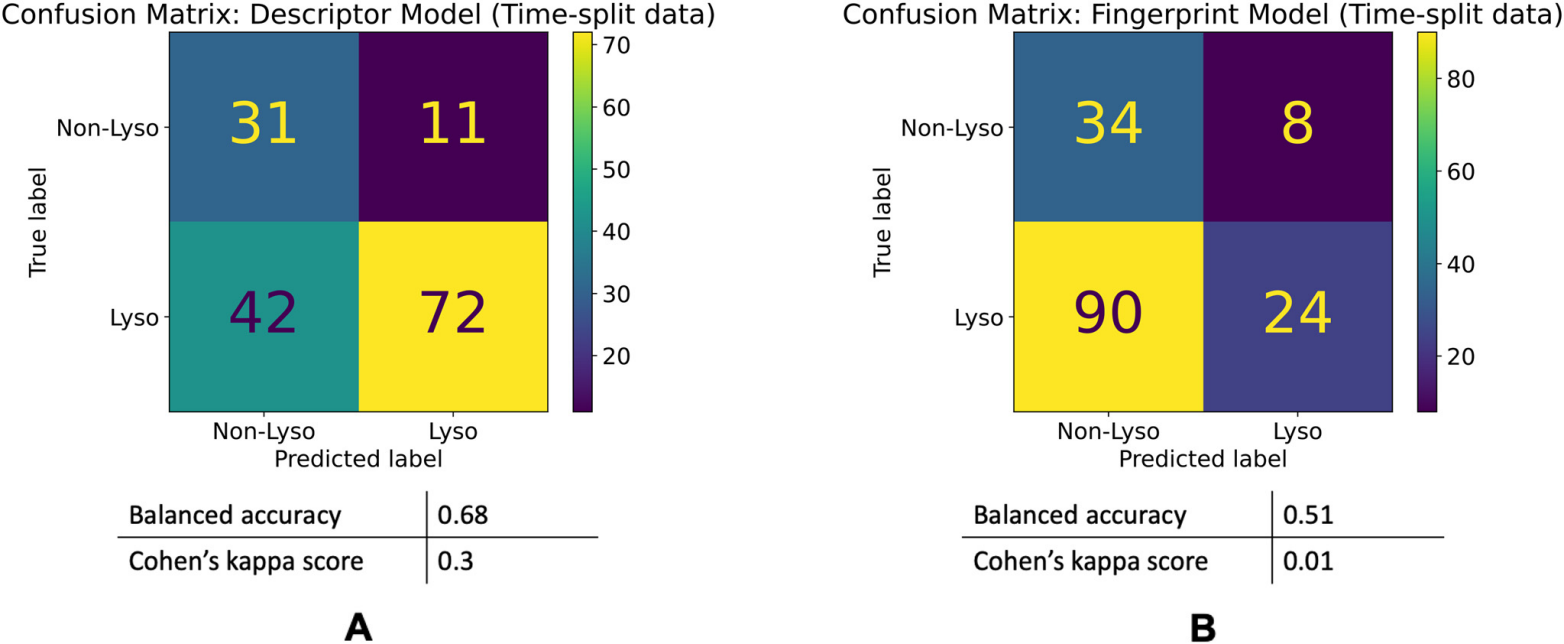
Model performances on the time-split dataset. Confusion matrices for the descriptor (A) and fingerprint (B) models' performances on the time-split dataset.

The descriptor model's balanced accuracy is 0.68 while its Cohen's kappa score is 0.3. The Fingerprint model's balanced accuracy is 0.51 and its Cohen's kappa is 0.01. The confusion matrices of these models' performances are shown in the Fig. 5.

#### External validation

**Fig. 6:**
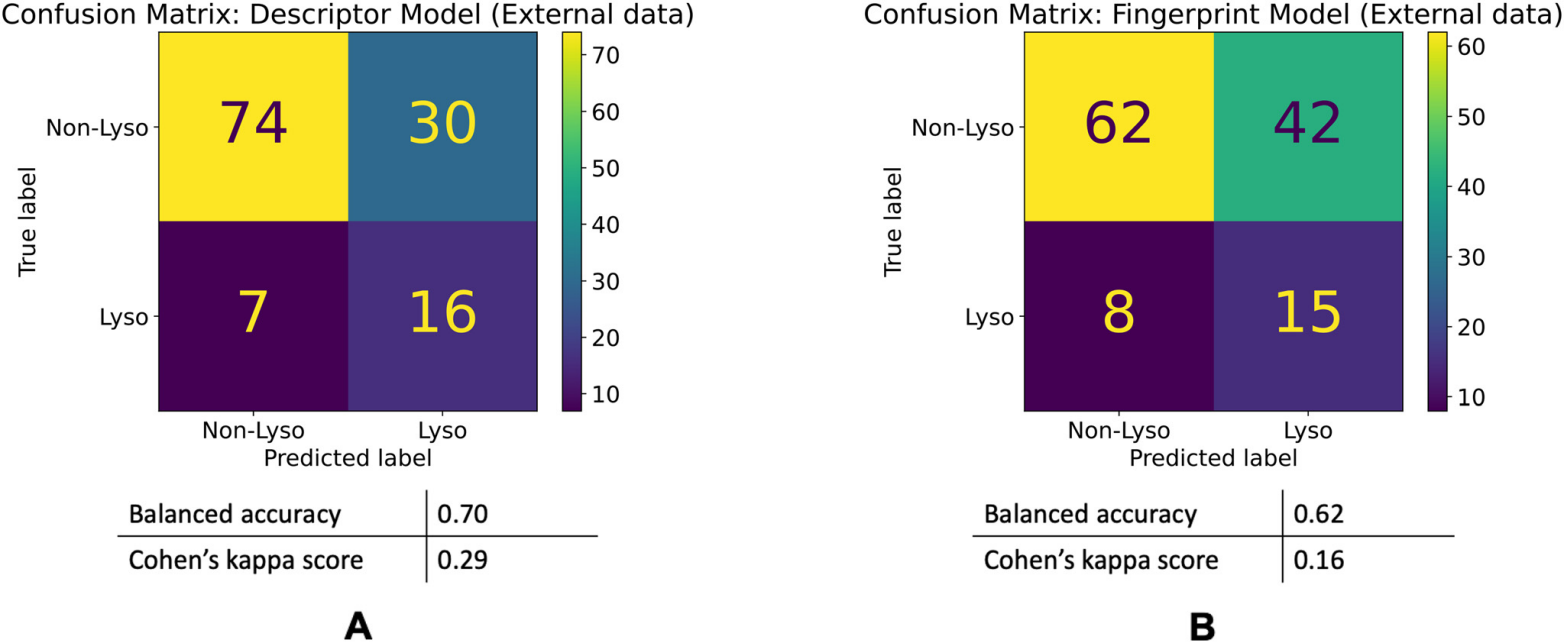
Model performances on the external dataset. Confusion matrices for the descriptor (A) and fingerprint (B) models' performances on the external dataset.

In addition, we performed an external validation of the models with purchased compounds. The selection process of choosing the compounds from the vendor is described in the Materials and methods section. Out of the 127 compounds in this dataset, in the CPA – 104 are non-lysosomotropic while the remaining 23 are lysosomotropic. The descriptor model's balanced accuracy is 0.70 while its Cohen's kappa score is 0.29. The fingerprint model's balanced accuracy is 0.62 and its Cohen's kappa is 0.16. The confusion matrices of these models' performances are shown in the Fig. 6.

#### SHAP analysis results

The SHAP summary plots are useful in visualizing the importance of individual input features. In the summary plots, Shapley values of each input feature across all the samples in the dataset are plotted together. Since these plots are bee swarm plots by default, the dots having the same Shapley value pile over each other. The input features are ranked higher based on their impact on the overall data. This ordering is based on the mean of the absolute Shapley values for each feature.

The color gradient from blue to red indicates the value of a feature from lower to higher. In our case, positive Shapley value correspond to the lysosomotropic class, and negative Shapley values to the non-lysosomotropic class.

**Fig. 7:**
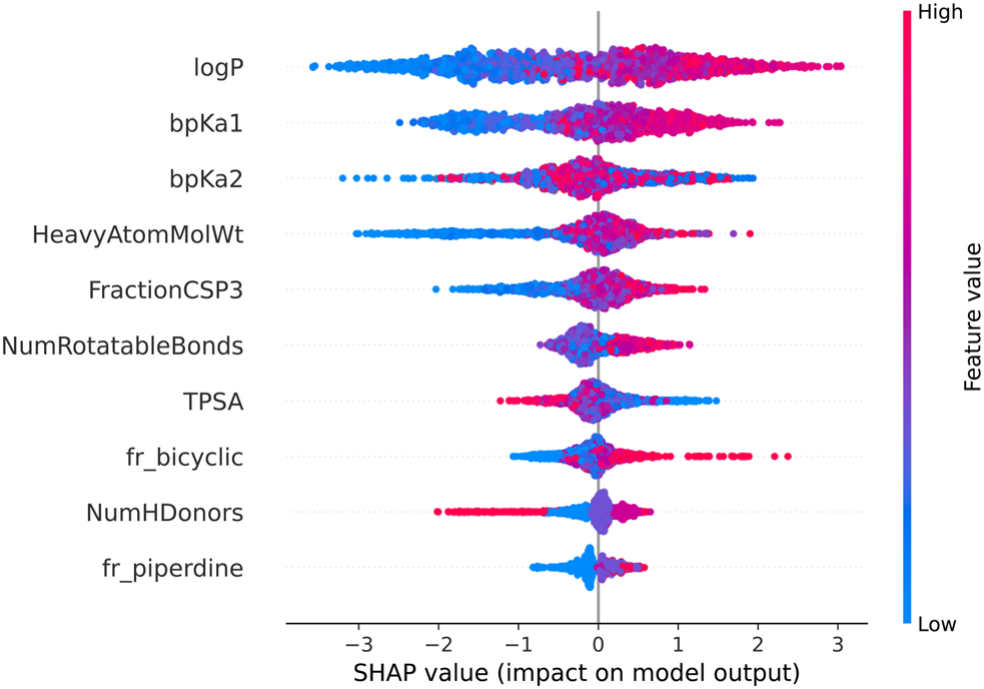
SHAP summary plot of the descriptor model's training dataset.

**Fig. 8:**
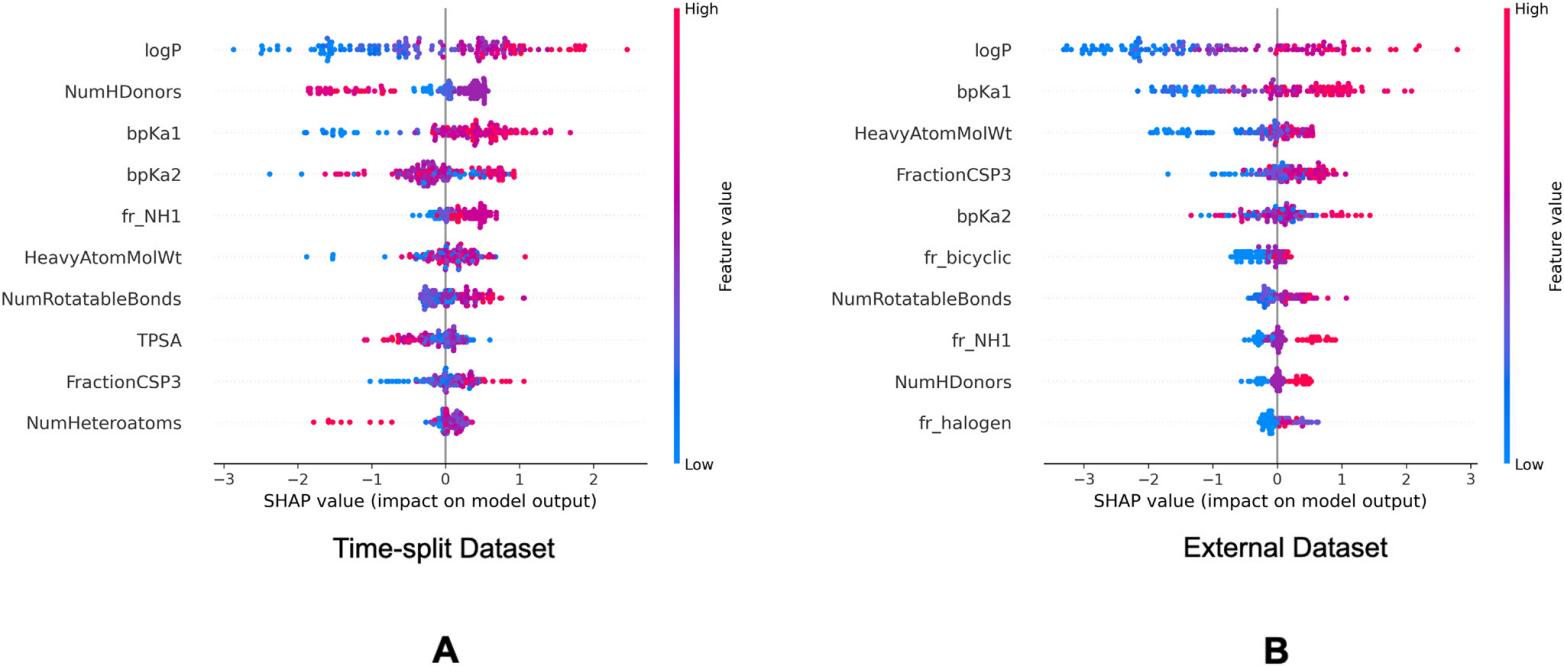
SHAP summary plots of the descriptor model on the validation datasets. Top 10 molecular descriptors found important in the descriptor model for the time-split dataset (A) and the external dataset (B).

Fig. 7 and 8 are the SHAP summary plots for the descriptor model on the training dataset and the validation datasets, respectively. These plots show the descriptors which are found important in each of the SHAP analysis.

SHAP dependence plots are scatter plots between a feature and their Shapley values. The dependence plots of top 10 features of the descriptor model for all the three datasets are present in the ESI† (Fig. S2–S4).

In the SHAP analysis of the training dataset, log *P* and bp*K*_a1_ are found as the most important descriptors. It can be observed in Fig. 7 that the higher log *P* and bp*K*_a1_ values contribute the model output towards the lysosomotropic class, and *vice versa*. Similar observations are noted in both of the SHAP analysis of the validations sets, the only exception being that the bp*K*_a1_ is the third most important descriptor in the time-split dataset.

Descriptor *fr_NH1* which describes the number of secondary amines, is found important in the SHAP analysis of the validations sets. Here, it is noted that the higher number of secondary amines have positive Shapley values indicating that they contribute model outputs to the lysosomotropic class.

Higher topological polar surface area (TPSA) is associated with poor cell membrane permeability. The inverse relationship between the TPSA values and their corresponding Shapley value indicates that the model outputs are driven towards non-lysosomotropic class when the TPSA of the compounds is higher. This can be justified if it is hypothesized that the non-lysosomotropic compounds have poor lysosome and/or cell membrane permeability.

Descriptor *HeavyAtomMolWt* calculates the average molecular weight of compounds while ignoring the hydrogen atoms. Across all three datasets, especially in the training and the external datasets, it can be observed that lower heavy atom molecular weights have negative Shapley values. Interestingly, the magnitude of negative Shapley values is higher than the positive Shapley values, indicating that the model finds lower molecular weights more important in classifying compounds as non-lysosomotropic.

The *X-FP* reports of the SHAP analysis of the fingerprint model of different datasets are present in the ESI.† Bit 2049, primarily encoding the sp^3^-hybridized carbon atom, is found important across all three datasets. When this bit is switched on – indicating the presence of the substructure encoded, the Shapley values are positive. This means that the presence of this substructure contributes the model prediction to the lysosomotropic class. Similarly, bit 3959, mainly encoding a secondary carbon across all the datasets, is found important in the training set and the external set. Positive Shapley value when this bit is switched on shows that presence of this substructure affects model predictions towards the lysosomotropic class.

Another bit encoding sp^3^ hybridized carbon is bit 1028 which encodes a carbon atom in the aliphatic ring. This bit is found important in the training dataset and the external dataset. Here too, presence of such substructure favors model predictions towards the lysosomotropic class. Interestingly, *FractionCSP3* is a descriptor which describes the fractions of sp^3^ hybridized carbons present in a compound, and this descriptor is found important in the SHAP analysis of the descriptor model of all the datasets. Thus, both of the models find the sp^3^ hybridized carbon substructures important and the therefore suggests predictions towards the lysosomotropic class in the presence of such substructures.

Bit 2715, depending on its neighboring groups, might be encoding a secondary amine. This bit is important across all the three datasets. Bit 3200 encoding an aliphatic nitrogen atom is important across all three datasets. For both of these cases, presence of these substructures would impact the model output towards the lysosomotropic class. This is in line with the finding that basic p*K*_a_ is consistently found as relevant descriptor in the SHAP analysis which is mostly driven by amine moieties.

The exemplar top bits and their substructures are shown in Table 1.

**Table 1:** Exemplary top bits and the key substructures they encode found important across different datasets by the fingerprint model. Presence of these substructures almost always influences model results towards lysosomotropism

Bits	Substructures	Found important in:	Shapley values
2049	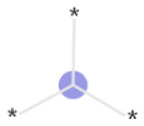	Training, time-split, external	Positive
3959	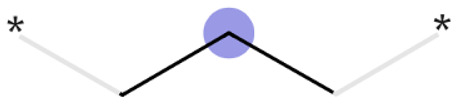	Training, external	Positive
1028	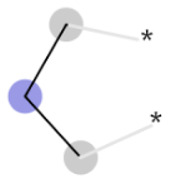	Training, external	Positive
2715	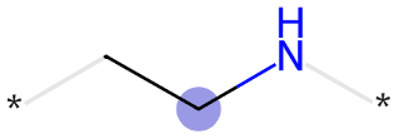	Training, time-split, external	Positive
3200	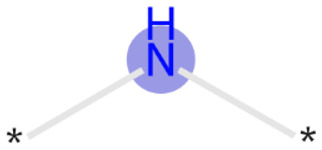	Training, time-split, external	Positive

#### Chemical structures and descriptor space similarities

**Fig. 9:**
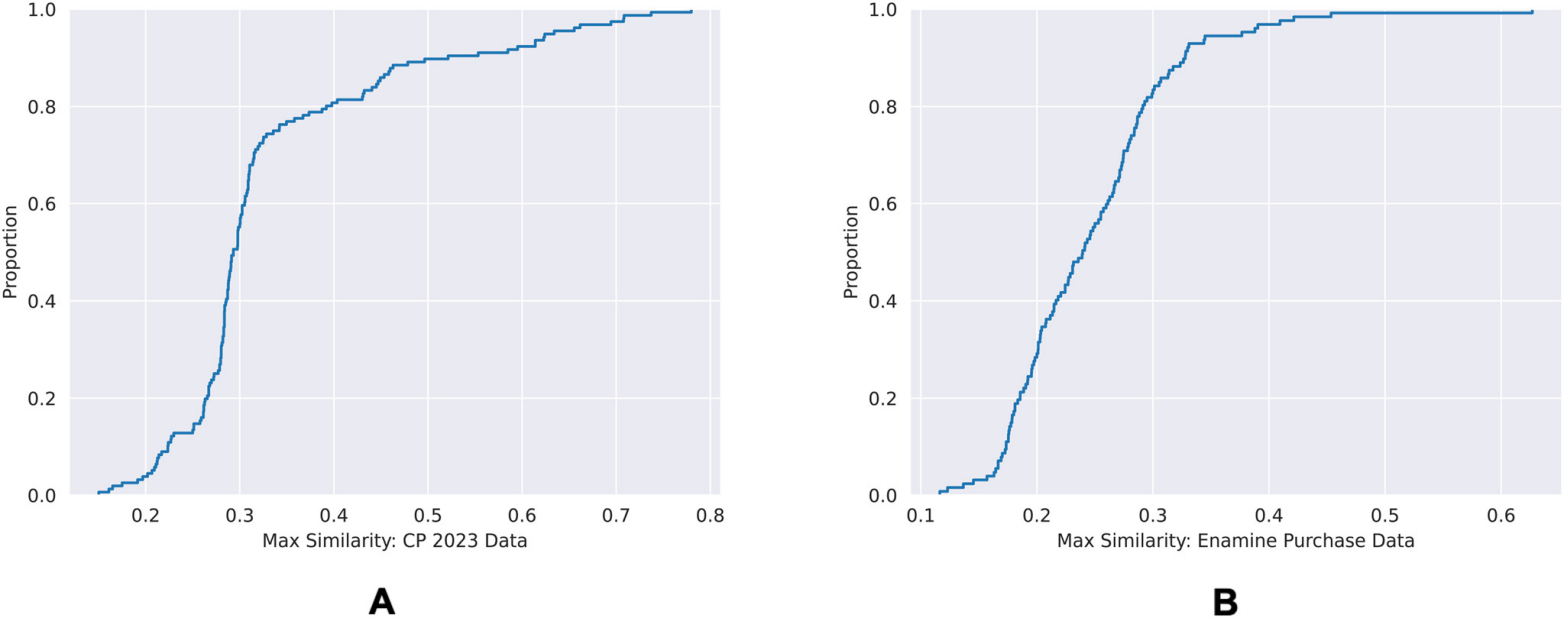
ECDF plots of the maximum chemical structure similarities of time-split dataset (A) and the external dataset (B) with the training dataset.

We performed chemical space similarity calculation, described in the Materials and methods section, to ensure the chemical structure diversity between the training dataset and both of the validation sets. The corresponding ECDF plots are shown in Fig. 9.

80% of the time-split dataset show a maximum Tanimoto similarity of less than 0.4 to the training set, whereas for the external dataset it is 0.3. This shows that the chemical spaces of both of the validation sets are diverse in comparison to the training set.

We also performed principal component analysis (PCA) of the input descriptors of the combined datasets. However, only 19% explained variance ratio was observed in the first three principal components.

## 3 Discussion

### 3.1 Lysosomotropic compounds tend to have higher log *P*

Even though log *P* was one of the two criteria for defining the PhysChem window used here, is the top descriptor in all 3 SHAP analyses of the descriptor model performed on the 3 data sets (training, time-split, and the external validation). All of these analyses show a common trend that higher log *P* values tend to have higher SHAP values and *vice versa*. Such relation can be interpreted such that with the higher log *P* values, the model favors the lysosomotropic class, and similarly with the lower log *P* values, the model instead favors the non-lysosomotropic class.

**Fig. 10:**
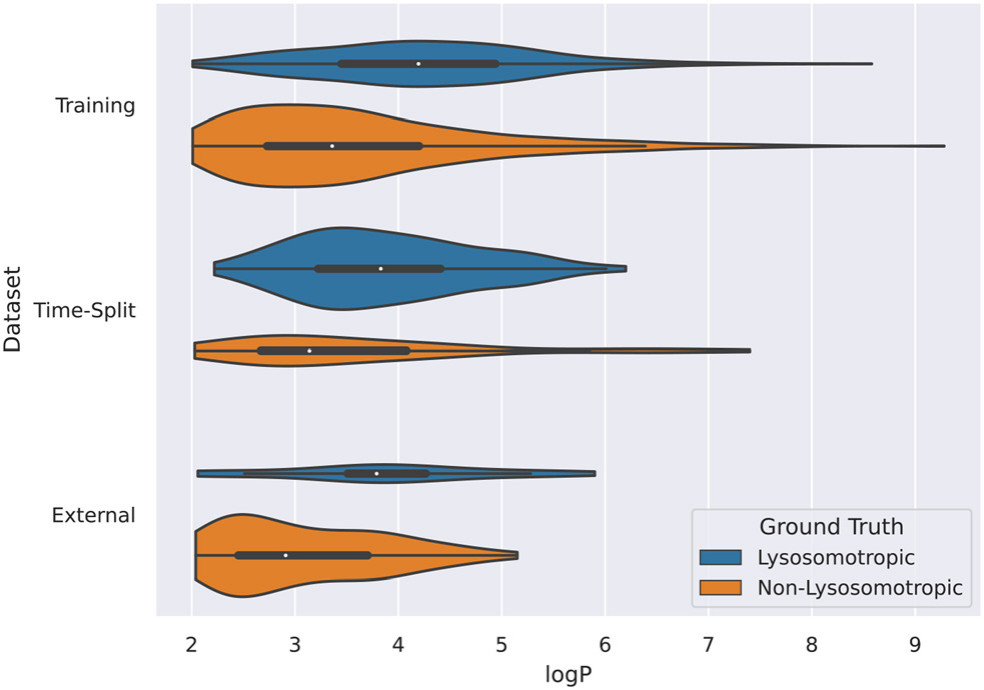
Violin plot of log *P* values across all datasets. The lysosomotropic classes are based on the cutoff of the compounds' lyso score. The violins are scaled based on the number of observations.

This relation between log *P* values and lysosomotropism can also be noticed in the violin plot of the original lysosomotropic class distribution *versus* the log *P* values (Fig. 10). Here, across all the three datasets, the lysosomotropic compounds tend to have higher log *P* values compared to the non-lysosomotropic ones.

### 3.2 Non-lysosomotropic compounds tend to have lower molecular weights

The descriptor *HeavyAtomMolWt* was found important in the SHAP analyses of all the datasets and it was noted that the lower molecular weights have negative Shapley values. This means such compounds are more likely to predicted non-lysosomotropic.

**Fig. 11:**
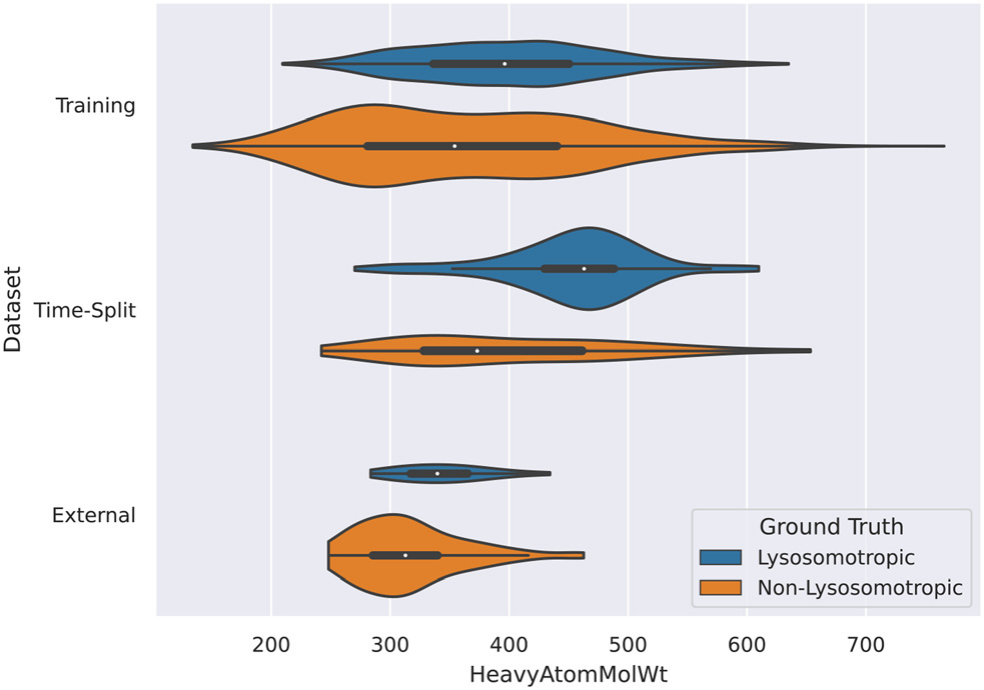
Violin plot of heavy atom molecular weight values across all datasets. The lysosomotropic classes are based on the cutoff of the compounds' lyso score. The violins are scaled based on the number of observations.

This relationship between molecular weight and non-lysosomotropism can be observed in the violin plot of the original lysosomotropic class distribution *versus* the molecular weight of the compounds (Fig. 11).

### 3.3 sp^3^-hybridized carbon atoms are found important

Different fingerprint bits encoding different sp^3^-hybridized carbon substructures are found important across all datasets by the fingerprint model. Furthermore, the descriptor *FractionCSP3* is found important by the descriptor model for all the datasets. The presence of sp^3^-hybridized carbon substructures is noted to impact the model output towards the lysosomotropic class, and *vice versa*, in all of the cases.

### 3.4 Basic moieties are found important

It is consistently found by the descriptor-based SHAP analysis as well as by the *X-FP* analysis that basic moieties are driving a compound to be lysosomotropic. Though we restricted our selected compounds to be within a certain PhysChem window (log *P* > 2, bp*K*_a_ between 6.2 and 11), this lead to a restriction of the entire data towards basic moieties. However, the XML still might not have been capable of identifying this bias, but they correctly identified the fact that basic moieties are relevant for lysosomotropism.

### 3.5 Key substructures

No key substructures which induce either lysosomotropism or non-lysosomotropism were found here. In the MMPA, we do not identify any dominant chemical substructures which determine lysosomotropism in our data. Similarly, while examining the fingerprint model with *X-FP*, the important bits usually encode sp^3^-hybridized carbon substructures and basic nitrogen substructures. We hypothesise that these substructures influence lysosomotropism by affecting the overall physicochemical properties of the compounds instead of causing specific chemical structurebased activity.

Many important bits encode substructures of the Morgan fingerprint radius of 0. This means that these substructures are single atoms, and thereby too unspecific to hypothesize any chemical structure-based activity.

### 3.6 The model performance

The descriptor model significantly outperforms the fingerprint model. Possible reasons for this initially unexpected drop in performance of the fingerprint model could be as follows.

First, as highlighted in the previous section, due to the absence of any key substructures inducing lysosomotropism, the substructures found important by the fingerprint model might not be specific enough to differentiate between lysosomotropic and non-lysosomotropic compounds. Furthermore, if lysosomotropism is mainly driven by log *P* and bp*K*_a_, then it will hard to find any substructure motifs that drive a log *P*/bp*K*_a_ change which in turn modulates lysosomotropism. This is due to the fact the very same (small, because it only considers the neighbors up to two bonds apart) substructure detected by a fingerprint can have very different log *P*/bp*K*_a_ values based on their decoration.

The chemical diversity of the training and the test sets form a challenge to the performance of the fingerprint model. This is especially pronounced when the presence or absence of the individual substructure (encoded as a bit in the fingerprint) has an impact on the biological readout, is influenced by the neighboring groups, the position in the molecule or by the stereochemistry, which is ignored by the fingerprints employed here.

The reasonable performance of the descriptor model compared to the fingerprint model further supports the notion that the lysosomotropism is primarily caused by physicochemical properties of a compound.

### 3.7 Concentration dependency of lysosomotropism

While investigating concentration dependency of lysosomotropism was not the focus of this work, we observed that not all the compounds show lysosomotropism even when tested at higher compound concentrations.

Out of 1369 non-lysosomotropic compounds present in the combined training set and time-split set, 682 of them were also tested at higher compound concentrations of either 30 μM and 50 μM in the CPA in addition to the standard 10 μM compound concentration. While approximately 40% of such compounds (277 compounds) show lysosomotropism at higher concentration, the remaining 405 compounds remain non-lysosomotropic. Moreover, out of these 405 compounds which stay non-lysosomotropic at both standard and higher concentrations, 255 of them are bioactive (induction >5).

Nadanaciva *et al.* also reported that the two non-lysosomotropic compounds present in the PhysChem window in their analysis, especially risperidone, did not show lysosomotropism even at the highest tested concentration of 150 μM.^15^

Further investigation in this area might offer insight on this observation.

### 3.8 Limitations

It is impractical to determine the log *P* values and the bp*K*_a_ values of the compounds in the wet-lab at a bigger scale, at least in an academic setup. Thus, in our study the values of these descriptors are predicted. These descriptors are influential in the study. First, the compound selection is based on the compounds' presence in the PhysChem window, which is the cross-section of the log *P* values and the bp*K*_a_ values. Second, these descriptors were found as the most important descriptors by the descriptor model. Therefore, our analyses might be affected due to the differences in the true and the predicted values of these descriptors.

While our internal data is a compilation of diverse compounds over many years, it cannot cover the full chemical space. Our models are trained on this finite data and the hypotheses derived from these results are therefore limited to this representation.

The lyso score of 75 or above, indicating biosimilarity, is a hard cut-off value to determine the lysosomotropic class of the compounds. The compounds around this cut-off value will be biosimilar, however, their lysosomotropic classes will be different. This could affect our analyses.

Lastly, the SHAP analyses do not show causation for the ground truth, rather they show what features were found important by a model in a dataset. Since our models' performances are limited, the SHAP analyses are also not definitive.

### 3.9 Conclusions

We used the morphological profiling data from the in-house cell painting assay (CPA) to identify lysosomotropism in small molecules. We applied the lyso score to quantitatively score lysosomotropism. We confirm that mere presence of a compound in the well-established cross-section window of log *P* and bp*K*_a_ values does not suffice it to be a lysosomotropic compound. By performing a MMPA, we were not able to detect key substructures that can be made responsible for a lysosomotropic effect.

Our ML models were trained on the internal dataset and tested on diverse validation sets. This ensured that these models' predictions and, by extension, the interpretable analyses done on them are general and not specific to the training set. These models revealed that the lysosomotropic effect is favored when the compounds have high lipophilicity, basicity, high number of basic amines and high number of sp^3^-hybridized carbons, and low TPSA.

### 3.10 LysoPredictor web app

A user-friendly web application to predict lysosomotropism by small compounds is available on the CzodrowskiLab website.^65^ This web application uses the descriptor model in the backend to make predictions.

## 4 Materials and methods

### 4.1 Data selection for MMPA, and model training and validation

The CPA protocol can be found in the methods section of Pahl *et al.* (2023).^66^ In total, roughly 13 000 compounds were measured in the cell painting assay out of which roughly 450 compounds are known lysosomotropic active as reported in literature. These compounds are used as reference system for comparison of the cell painting profiles of the remaining compounds. Out the overall 13 000 tested compounds, 738 compounds are inside the PhysChem window and lysosomotropic. But there are 1327 compounds are inside the PhysChem which are not lysosomotropic. A filtering strategy was employed on the internal CP data set till 2022. 10 μM compound concentration was selected. Toxic compounds and those flagged with purity alerts were excluded. Compounds with heavy atom count exceeding 50 were removed. Compounds whose calculated log *P* and bp*K*_a1_ values were empty were removed. Final selection was then made on the compounds present in the PhysChem window – log *P* value greater than 2 and bp*K*_a1_ value greater than 6.2 and less than 11. ap*K*_a1_ and ap*K*_a2_ were removed since they were redundant in the context of this study. In total, 2065 compounds were available for MMPA, and model training and cross validation.

The bp*K*_a1_ limit of 6.5 from literature was lowered to 6.2 to enable the inclusion of a larger group of lysosomotropic compounds (53 compounds) present in the bp*K*_a1_ range between 6.5 and 6.2.

Same steps were repeated to obtain compounds for the time-split validation set on the 2023 dataset.

Details about selected compounds from the time-split validation and the training data can be found here: ref. 34, 37, 39, 67 and 68.

The corresponding code for the filtering strategy is available on GitHub.^69^

### 4.2 The mmpdb algorithm

The mmpdb algorithm (version 2) published by Dalke *et al.* (2018),^45^ which is freely available from the RDKit repository, was employed with default parameters. The program takes a set of SMILES of the compounds under analysis as input and outputs SMIRKS describing the molecular transformation for each MMP identified.^51^ In brief, it consists of a three-step-procedure: firstly, the compounds in the data set are decomposed into fragments following a well-defined protocol to derive all possible constant and variable parts, secondly all fragments are indexed and systematically compared to identify common substructures.^46,51^ Lastly, the transformation rules are derived by evaluating all the MMPs with the same transformation (same variable A and B) at a specific environment radius and aggregation of the associated property changes.^70^

### 4.3 Explainable machine learning

#### Molecular descriptors and molecular fingerprints calculations

All the molecular descriptors, except log *P* and bp*K*_a_, were calculated by the RDKit. Log *P* and bp*K*_a_ were calculated by ChemAxon Marvin *cxcalc* version *22.22.0* (https://www.chemaxon.com).

All the molecular fingerprints were generated by the RDKit. The Morgan fingerprints were calculated as bit vectors and the option to save the bit info was enabled to perform the SHAP analysis of the substructures using *X-FP*.

#### Modelling

The corresponding code for the modelling and cross-validation is available on GitHub.^69^

#### Chemical space similarity calculation

The Morgan fingerprint of radius 4 were used to generate structural information of the compounds. Tanimoto similarity was used to compare fingerprints of two compounds, with the scale of similarity between 0 and 1 where 0 indicates no similarity and 1 indicates complete similarity.

For each compound present in a query dataset, chemical structure similarity against all of the compounds in the training dataset was calculated. The compound present in the training data which is structurally most similar to the query compound would therefore give the highest similarity score, and this score would be the query compound's maximum similarity score against the training dataset.

By plotting the maximum similarity scores of all the compounds of the query dataset as an ECDF plot, the percentage similarity to the training dataset can be observed.

#### External validation

The *Liquid Stock Collection* from February 2023 comprising approximately 270 thousand compounds from the vendor Enamine was chosen. A compound standardization was performed which involved removal of compounds having a minimum heavy atom count of 20 and maximum heavy atom count of 50, *MedChem* filters, structure canonicalization, and duplicate removal.^71^ Next, common compounds present in the internal MPI dataset were removed. Log *P* and bp*K*_a_ were calculated by ChemAxon Marvin *cxcalc* version *22.22.0* (https://www.chemaxon.com) – the compounds present outside the PhysChem window were removed. To remove promiscuous compounds from the data in the following step, filtering proposed by Novartis Institutes of BioMedical Research (NIBR), which includes filters for the PAINS filter families A and B, was performed.^72,73^

The purchased compounds have at least a purity of 90 percent, measured by liquid chromatography–mass spectrometry (LC–MS). More details can be found in the ESI.†

Maximum similarity calculation was performed against the internal MPI dataset and diverse compounds were short-listed. The original lysosomotropic class ratio present in the training dataset (65% non-lysosomotropic and 35% lysosomotropic) was aimed to be maintained, therefore, 127 compounds where 81 as non-lysosomotropic and 46 as lysosomotropic compounds predicted by the descriptor model were short-listed and ordered. For these 127 compounds, the fingerprint model classified 70 as non-lysosomotropic and 57 as lysosomotropic.

## List of abbreviations

bp*K*_a_: Basic p*K*_a_
clog *P*: Calculated log *P*
CP: Cell painting
CPA: Cell painting assay
LC–MS: Liquid chromatography-mass spectrometry
log *P*: Octanol/water-partition coefficient
LTR: LysoTracker Red DND-99
ML: Machine learning
MMP: Matched molecular pair
MMPA: Matched molecular pair analysis
PCA: Principal component analysis
QSAR: Quantitative structure activity relationship
SAG: Smoothened agonist
SAR: Structure activity relationship
SARS-CoV-2: Severe acute respiratory syndrome coronavirus 2
SHAP: Shapley additive explanations
SMILES: Simplified molecular input line entry system
SPR: Structure property relationship
TPSA: Topological polar surface area
XGBoost: Extreme gradient boosting
XML: Explainable machine learning

## Conflicts of interest

The authors declare no conflicts of interest.

## Supplementary Material

MD-015-D4MD00107A-s001

## References

[cit1] de Duve C., de Barsy T., Poole B., Trouet A., Tulkens P., van Hoof F. (1974). Lysosomotropic Agents. Biochem. Pharmacol..

[cit2] Pisonero-Vaquero S., Medina D. L. (2017). Lysosomotropic Drugs: Pharmacological Tools to Study Lysosomal Function. Curr. Drug Metab..

[cit3] Schneidewind T., Brause A., Schölermann B., Sievers S., Pahl A., Sankar M. G., Winzker M., Janning P., Kumar K., Ziegler S., Waldmann H. (2021). Combined morphological and proteome profiling reveals target-independent impairment of cholesterol homeostasis. Cell Chem. Biol..

[cit4] Kuzu O. F., Toprak M., Noory M. A., Robertson G. P. (2017). Effect of lysosomotropic molecules on cellular homeostasis. Pharmacol. Res..

[cit5] Blaess M., Kaiser L., Sauer M., Csuk R., Deigner H.-P. (2020). COVID-19/SARS-CoV-2 Infection: Lysosomes and Lysosomotropism Implicate New Treatment Strategies and Personal Risks. Int. J. Mol. Sci..

[cit6] Vincent M. J., Bergeron E., Benjannet S., Erickson B. R., Rollin P. E., Ksiazek T. G., Seidah N. G., Nichol S. T. (2005). Chloroquine is a potent inhibitor of SARS coronavirus infection and spread. Virol. J..

[cit7] Norinder U., Tuck A., Norgren K., Kos V. M. (2020). Existing highly accumulating lysosomotropic drugs with potential for repurposing to target COVID-19. Biomed. Pharmacother..

[cit8] Keyaerts E., Vijgen L., Maes P., Neyts J., Ranst M. V. (2004). In vitro inhibition of severe acute respiratory syndrome coronavirus by chloroquine. Biochem. Biophys. Res. Commun..

[cit9] Devaux C. A., Rolain J. M., Colson P., Raoult D. (2020). New insights on the antiviral effects of chloroquine against coronavirus: what to expect for COVID-19?. Int. J. Antimicrob. Agents.

[cit10] Tummino T. A., Rezelj V. V., Fischer B., Fischer A., O'Meara M. J., Monel B., Vallet T., White K. M., Zhang Z., Alon A., Schadt H., O’Donnell H. R., Lyu J., Rosales R., McGovern B. L., Rathnasinghe R., Jangra S., Schotsaert M., Galarneau J.-R., Krogan N. J., Urban L., Shokat K. M., Kruse A. C., García-Sastre A., Schwartz O., Moretti F., Vignuzzi M., Pognan F., Shoichet B. K. (2021). Drug-induced phospholipidosis confounds drug repurposing for SARS-CoV-2. Science.

[cit11] Henao-Restrepo A. M., Pan H., Peto R., Preziosi M. P., Sathiyamoorthy V. (2022). *et al.* Remdesivir and three other drugs for hospitalised patients with COVID-19: final results of the WHO Solidarity randomised trial and updated meta-analyses. Lancet.

[cit12] Chloroquine or Hydroxychloroquine and/or Azithromycin National Institutes of Health (NIH) COVID-19 Treatment Guidelines. Accessed: 2023-02-25

[cit13] Table. Chloroquine or Hydroxychloroquine and/or Azithromycin: Selected Clinical Data National Institutes of Health (NIH) COVID-19 Treatment Guidelines. Accessed: 2023-02-25

[cit14] Marceau F., Bawolak M. T., Lodge R., Bouthillier J., Gagné-Henley A., Gaudreault R. C., Morissette G. (2012). Cation trapping by cellular acidic compartments: Beyond the concept of lysosomotropic drugs. Toxicol. Appl. Pharmacol..

[cit15] Nadanaciva S., Lu S., Gebhard D. F., Jessen B. A., Pennie W. D., Will Y. (2011). A high content screening assay for identifying lysosomotropic compounds. Toxicol. In Vitro.

[cit16] Zhitomirsky B., Assaraf Y. G. (2015). Lysosomal sequestration of hydrophobic weak base chemotherapeutics triggers lysosomal biogenesis and lysosome-dependent cancer multidrug resistance. Onco Targets Ther.

[cit17] Lu S., Sung T., Lin N., Abraham R. T., Jessen B. A. (2017). Lysosomal adaptation: How cells respond to lysosomotropic compounds. PLoS One.

[cit18] Ufuk A., Assmus F., Francis L., Plumb J., Damian V., Gertz M., Houston J. B., Galetin A. (2017). In Vitro and in Silico Tools To Assess Extent of Cellular Uptake and Lysosomal Sequestration of Respiratory Drugs in Human Alveolar Macrophages. Mol. Pharmaceutics.

[cit19] Schmitt M. V., Lienau P., Fricker G., Reichel A. (2019). Quantitation of Lysosomal Trapping of Basic Lipophilic Compounds Using In Vitro Assays and In Silico Predictions Based on the Determination of the Full pH Profile of the Endo−/Lysosomal System in Rat Hepatocytes. Drug Metab. Dispos..

[cit20] Norinder U., Kos V. M. (2019). QSAR models for predicting five levels of cellular accumulation of lysosomotropic macrocycles. Int. J. Mol. Sci..

[cit21] Hu H., Tjaden A., Knapp S., Antolin A. A., Müller S. (2023). A machine learning and live-cell imaging tool kit uncovers small molecules induced phospholipidosis. Cell Chem. Biol..

[cit22] Ohkuma S., Poole B. (1981). Cytoplasmic vacuolation of mouse peritoneal macrophages and the uptake into lysosomes of weakly basic substances. J. Cell Biol..

[cit23] Bray M.-A., Singh S., Han H., Davis C. T., Borgeson B., Hartland C., Kost-Alimova M., Gustafsdottir S. M., Gibson C. C., Carpenter A. E. (2016). Cell Painting, a high-content image-based assay for morphological profiling using multiplexed fluorescent dyes. Nat. Protoc..

[cit24] Chen H., Engkvist O., Wang Y., Olivecrona M., Blaschke T. (2018). The rise of deep learning in drug discovery. Drug Discovery Today.

[cit25] Schneider P., Walters W. P., Plowright A. T., Sieroka N., Listgarten J., Goodnow R. A., Fisher J., Jansen J. M., Duca J. S., Rush T. S. (2019). *et al.* Rethinking drug design in the Artificial Intelligence Era. Nat. Rev. Drug Discovery.

[cit26] Ribeiro M. T. , Singh S. and Guestrin C., Proc. 22nd ACM SIGKDD Int. Conf. on Knowledge Discovery and Data Mining, 2016, pp. 1135–1144

[cit27] Lundberg S. M. and Lee S. I., A unified approach to interpreting model predictions, Advances in Neural Information Processing Systems, 2017, pp. 4766–4775

[cit28] Rodríguez-Pérez R., Bajorath J. (2020). Interpretation of compound activity predictions from complex machine learning models using local approximations and shapley values. J. Med. Chem..

[cit29] Jiménez-Luna J., Skalic M., Weskamp N., Schneider G. (2021). Coloring Molecules with Explainable Artificial Intelligence for Preclinical Relevance Assessment. J. Chem. Inf. Model..

[cit30] Harren T., Matter H., Hessler G., Rarey M., Grebner C. (2022). Interpretation of Structure–Activity Relationships in Real-World Drug Design Data Sets Using Explainable Artificial Intelligence. J. Chem. Inf. Model..

[cit31] Gustafsdottir S. M., Ljosa V., Sokolnicki K. L., Wilson J. A., Walpita D., Kemp M. M., Seiler K. P., Carrel H. A., Golub T. R., Schreiber S. L., Clemons P. A., Carpenter A. E., Shamji A. F. (2013). Multiplex Cytological Profiling Assay to Measure Diverse Cellular States. PLoS One.

[cit32] Gally J.-M., Pahl A., Waldmann H. (2020). Identifying bioactivity of pseudo-natural products using the Cell Painting assay. ARKIVOC.

[cit33] Schölermann B., Bonowski J., Grigalunas M., Burhop A., Xie Y., Hoock J. G. F., Liu J., Dow M., Nelson A., Nowak C., Pahl A., Sievers S., Ziegler S. (2022). Identification of Dihydroorotate Dehydrogenase Inhibitors Using the Cell Painting Assay. ChemBioChem.

[cit34] Christoforow A., Wilke J., Binici A., Pahl A., Ostermann C., Sievers S., Waldmann H. (2019). Design, Synthesis, and Phenotypic Profiling of Pyrano-Furo-Pyridone Pseudo Natural Products. Angew. Chem., Int. Ed..

[cit35] Schneidewind T., Brause A., Pahl A., Burhop A., Mejuch T., Sievers S., Waldmann H., Ziegler S. (2020). Morphological Profiling Identifies a Common Mode of Action for Small Molecules with Different Targets. ChemBioChem.

[cit36] Laraia L. (2020). *et al.*, Image-Based Morphological Profiling Identifies a Lysosomotropic, Iron-Sequestering Autophagy Inhibitor. Angew. Chem., Int. Ed..

[cit37] Foley D. J., Zinken S., Corkery D., Laraia L., Pahl A., Wu Y.-W., Waldmann H. (2020). Phenotyping Reveals Targets of a Pseudo-Natural-Product Autophagy Inhibitor. Angew. Chem., Int. Ed..

[cit38] Kumar K., Waldmann H. (2009). Synthesis of Natural Product Inspired Compound Collections. Angew. Chem., Int. Ed..

[cit39] Grigalunas M., Burhop A., Zinken S., Pahl A., Gally J.-M., Wild N., Mantel Y., Sievers S., Foley D. J., Scheel R., Strohmann C., Antonchick A. P., Waldmann H. (2021). Natural product fragment combination to performance-diverse pseudo-natural products. Nat. Commun..

[cit40] Imatinib DrugBank. Accessed: 2023-03-07

[cit41] Toremifene DrugBank. Accessed: 2023-03-07

[cit42] Clozapine DrugBank. Accessed: 2023-03-07

[cit43] Kenney P. W. and Sadowski J., Structure Modification in Chemical Databases, Wiley-VCH, 2004, p. 493

[cit44] Papadatos G., Alkarouri M., Gillet V. J., Willett P., Kadirkamanathan V., Luscombe C. N., Bravi G., Richmond N. J., Pickett S. D., Hussain J., Pritchard J. M., Cooper A. W., MacDonald S. J. (2010). Lead Optimization Using Matched Molecular Pairs: Inclusion of Contextual Information for Enhanced Prediction of hERG Inhibition, Solubility, and Lipophilicity. J. Chem. Inf. Model..

[cit45] Dalke A., Hert J., Kramer C. (2018). Mmpdb: An Open-Source Matched Molecular Pair Platform for Large Multiproperty Data Sets. J. Chem. Inf. Model..

[cit46] Wassermann A. M., Dimova D., Iyer P., Bajorath J. (2012). Advances in Computational Medicinal Chemistry: Matched Molecular Pair Analysis. Drug Dev. Res..

[cit47] Dossetter A. G., Griffen E. J., Leach A. G. (2013). Matched Molecular Pair Analysis in Drug Discovery. Drug Discovery Today.

[cit48] Tyrchan C., Evertsson E. (2017). Matched Molecular Pair Analysis in Short: Algorithms, Applications and Limitations. Comput. Struct. Biotechnol. J..

[cit49] Griffen E., Leach A. G., Robb G. R., Warner D. J. (2011). Matched molecular pairs as a medicinal chemistry tool. J. Med. Chem..

[cit50] Awale M., Hert J., Guasch L., Riniker S., Kramer C. (2021). The Playbooks of Medicinal Chemistry Design Moves. J. Chem. Inf. Model..

[cit51] Hussain J., Rea C. (2010). Computationally efficient algorithm to identify matched molecular pairs (MMPs) in large data sets. J. Chem. Inf. Model..

[cit52] Rogers D., Hahn M. (2010). Extended-Connectivity Fingerprints. J. Chem. Inf. Model..

[cit53] Morgan H. L. (1965). The Generation of a Unique Machine Description for Chemical Structures-A Technique Developed at Chemical Abstracts Service. J. Chem. Doc..

[cit54] Gedeck P., Rohde B., Bartels C. (2006). QSAR – How Good Is It in Practice? Comparison of Descriptor Sets on an Unbiased Cross Section of Corporate Data Sets. J. Chem. Inf. Model..

[cit55] Todeschini R. and Consonni V., Molecular Descriptors for Cheminformatics, Wiley-VCH, 2009

[cit56] rdkit/rdkit: 2022_09_5 (Q3 2022) Release, 2023

[cit57] Bramer M. , Principles of Data Mining, Springer, London, 2013, pp. 121–136

[cit58] Chen T. and Guestrin C., Proc. 22nd ACM SIGKDD Int. Conf. on Knowledge Discovery and Data Mining, 2016, pp. 785–794

[cit59] Akiba T. , Sano S., Yanase T., Ohta T. and Koyama M., Proc. 25th ACM SIGKDD Conf., 2019

[cit60] Buitinck L. , Louppe G., Blondel M., Pedregosa F., Mueller A., Grisel O., Niculae V., Prettenhofer P., Gramfort A., Grobler J., Layton R., VanderPlas J., Joly A., Holt B. and Varoquaux G., ECML PKDD Workshop: Languages for Data Mining and Machine Learning, 2013, pp. 108–122

[cit61] Shapley L. S. , Contributions to the Theory of Games (AM-28), Princeton University Press, 1953, vol. 2, pp. 307–318

[cit62] Humer C., Heberle H., Montanari F., Wolf T., Huber F., Henderson R., Heinrich J., Streit M. (2022). ChemInformatics Model Explorer (CIME): exploratory analysis of chemical model explanations. J. Cheminf..

[cit63] Lundberg S. M., Erion G., Chen H., DeGrave A., Prutkin J. M., Nair B., Katz R., Himmelfarb J., Bansal N., Lee S. I. (2020). From local explanations to global understanding with explainable AI for trees. Nat. Mach. Intell..

[cit64] Tandon A. and Baltruschat M., X-FP: eXplainable FingerPrints X-FP GitHub repository. Accessed: 2023-11-30

[cit65] Lysosomotropism Predictor WebApp CzodrowskiLab Homepage. Accessed: 2023-10-24

[cit66] Pahl A., Schölermann B., Lampe P., Rusch M., Dow M., Hedberg C., Nelson A., Sievers S., Waldmann H., Ziegler S. (2023). Morphological subprofile analysis for bioactivity annotation of small molecules. Cell Chem. Biol..

[cit67] Zimmermann S., Akbarzadeh M., Otte F., Strohmann C., Sankar M. G., Ziegler S., Pahl A., Sievers S., Kumar K. (2019). A Scaffold-Diversity Synthesis of Biologically Intriguing Cyclic Sulfonamides. Chem. – Eur. J..

[cit68] Liu J., Cremosnik G. S., Otte F., Pahl A., Sievers S., Strohmann C., Waldmann H. (2021). Design, Synthesis, and Biological Evaluation of Chemically and Biologically Diverse Pyrroquinoline Pseudo Natural Products. Angew. Chem., Int. Ed..

[cit69] Lysosomotropic Project GitHub Repo CzodrowskiLab Lyso Project Open GitHub repository. Accessed: 2023-11-30

[cit70] Awale M., Riniker S., Kramer C. (2020). Matched Molecular Series Analysis for ADME Property Prediction. J. Chem. Inf. Model..

[cit71] Pahl A. , Jupy Tools, version 1.0.0, 2022

[cit72] Schneider N. and Schuffenhauer A., NIBR Substructure Filters Python Script RDKit Contrib NIBRSubstructureFilters GitHub repository. Accessed: 2023-11-30

[cit73] Schuffenhauer A. (2020). *et al.* Evolution of Novartis' Small Molecule Screening Deck Design. J. Med. Chem..

