# Supplementary material for: Identification of lysosomotropism using explainable machine learning and morphological profiling cell painting data
Source: RSC Med Chem. 2024 May 24;15(8):2677–91. doi: 10.1039/d4md00107a (PMC11324048; doi:10.1039/d4md00107a)
Supplement: MD-015-D4MD00107A-s001 [file MD-015-D4MD00107A-s001.pdf]

Electronic Supplementary Material (ESI) for RSC Medicinal Chemistry.
This journal is © The Royal Society of Chemistry 2024

Supplementary Information: Identification of Lysosomotropism using Explainable Machine Learning and Morphological Profiling Cell Painting Data

Aishvarya Tandon, Anna Santura, Herbert Waldmann*,
Axel Pahl*, Paul Czodrowski*

2023

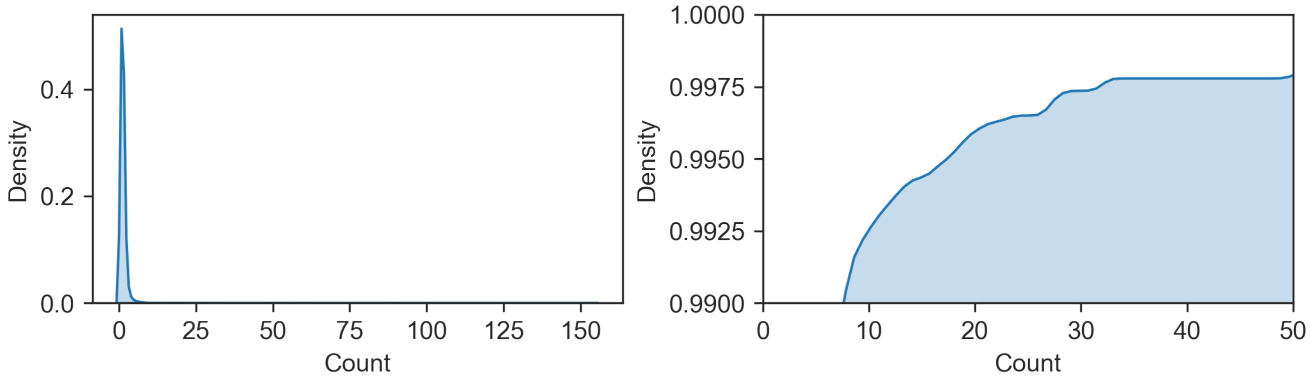

Figure S1: MMPA: (Cumulative) Density distribution of counts (number of occurrence) of transformations.

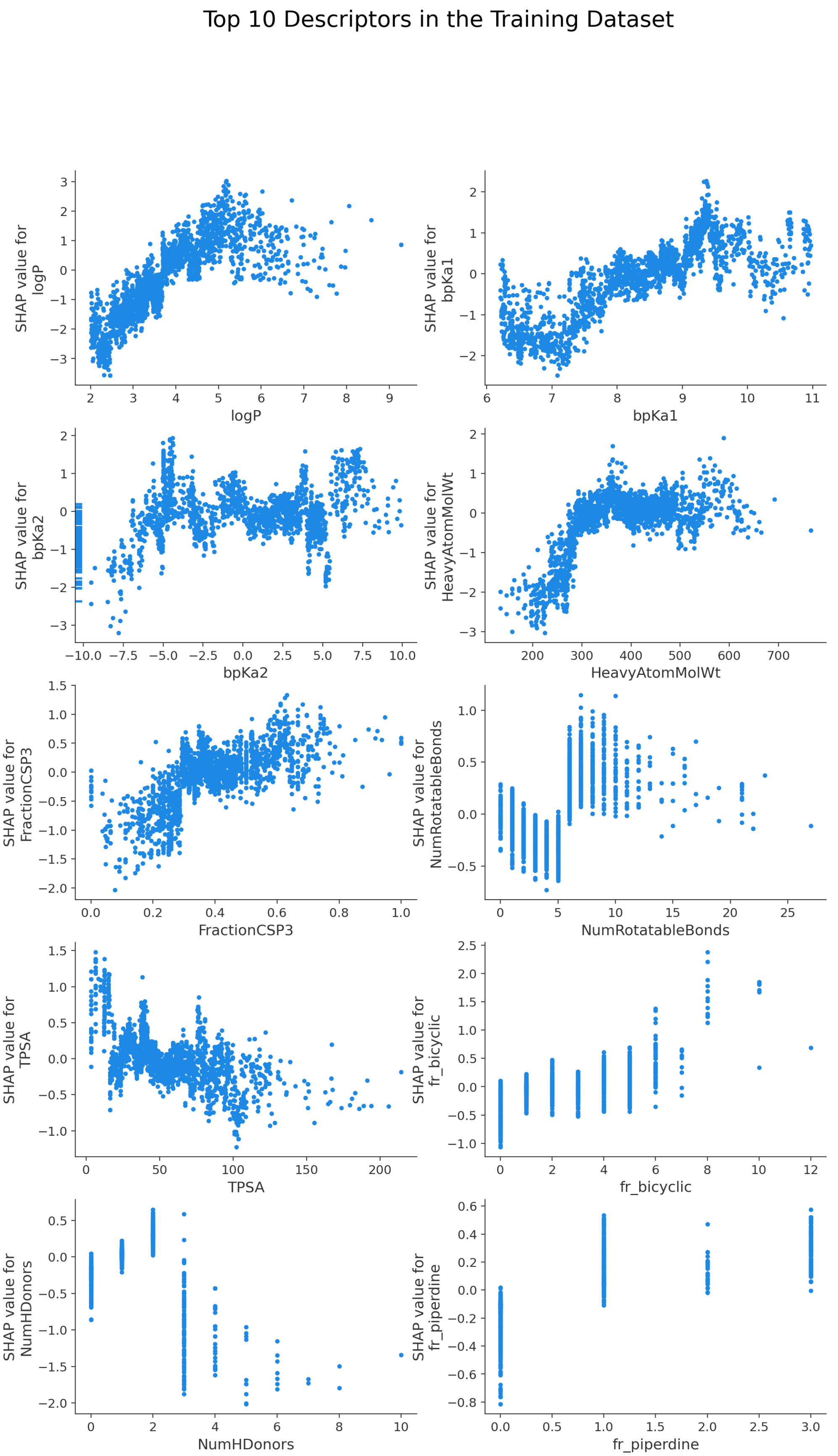

Figure S2: Descriptor model: Top 10 descriptors' Dependence Plots in the training dataset.

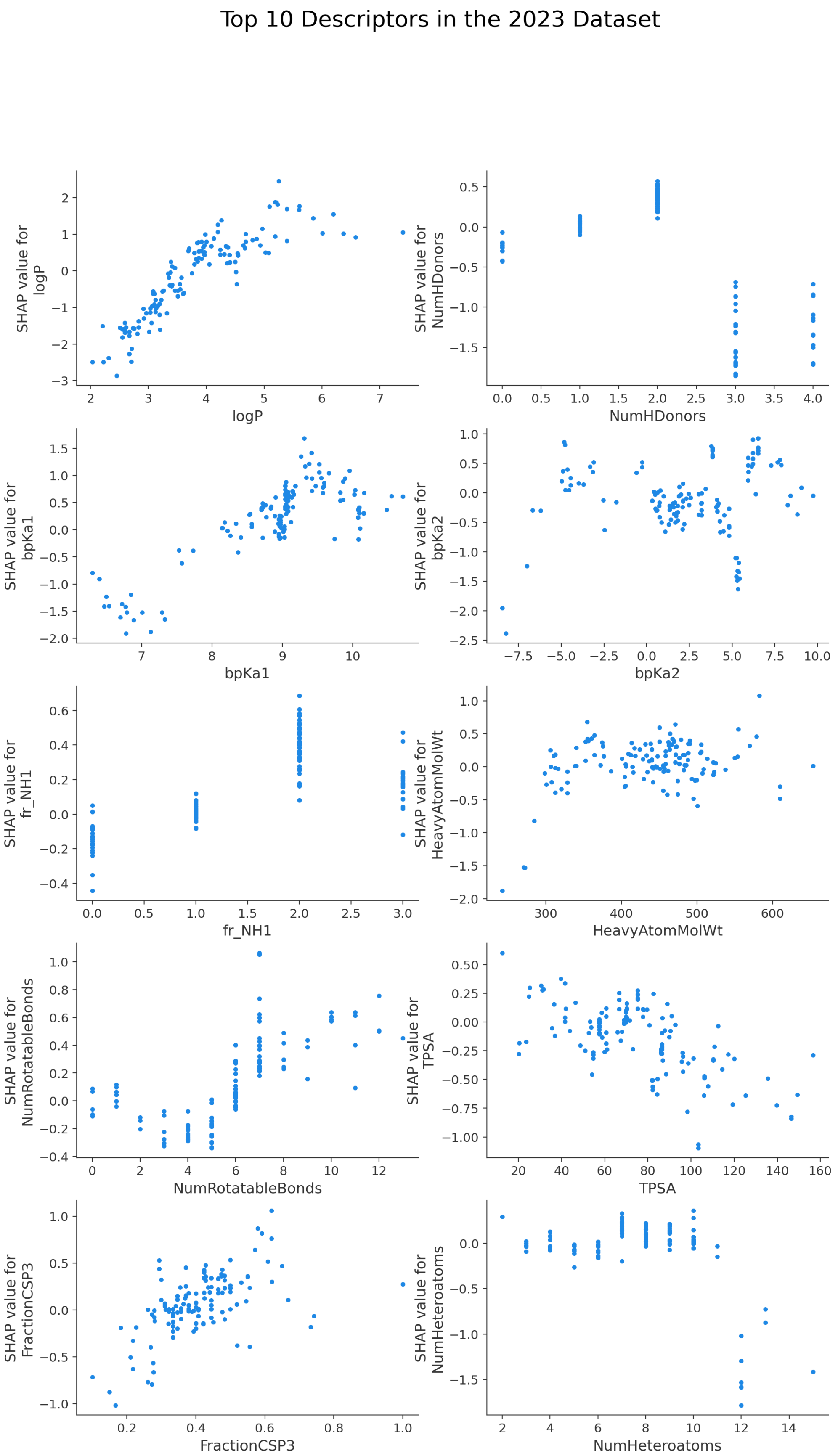

Figure S3: Descriptor model: Top 10 descriptors' Dependence Plots in the time-split dataset.

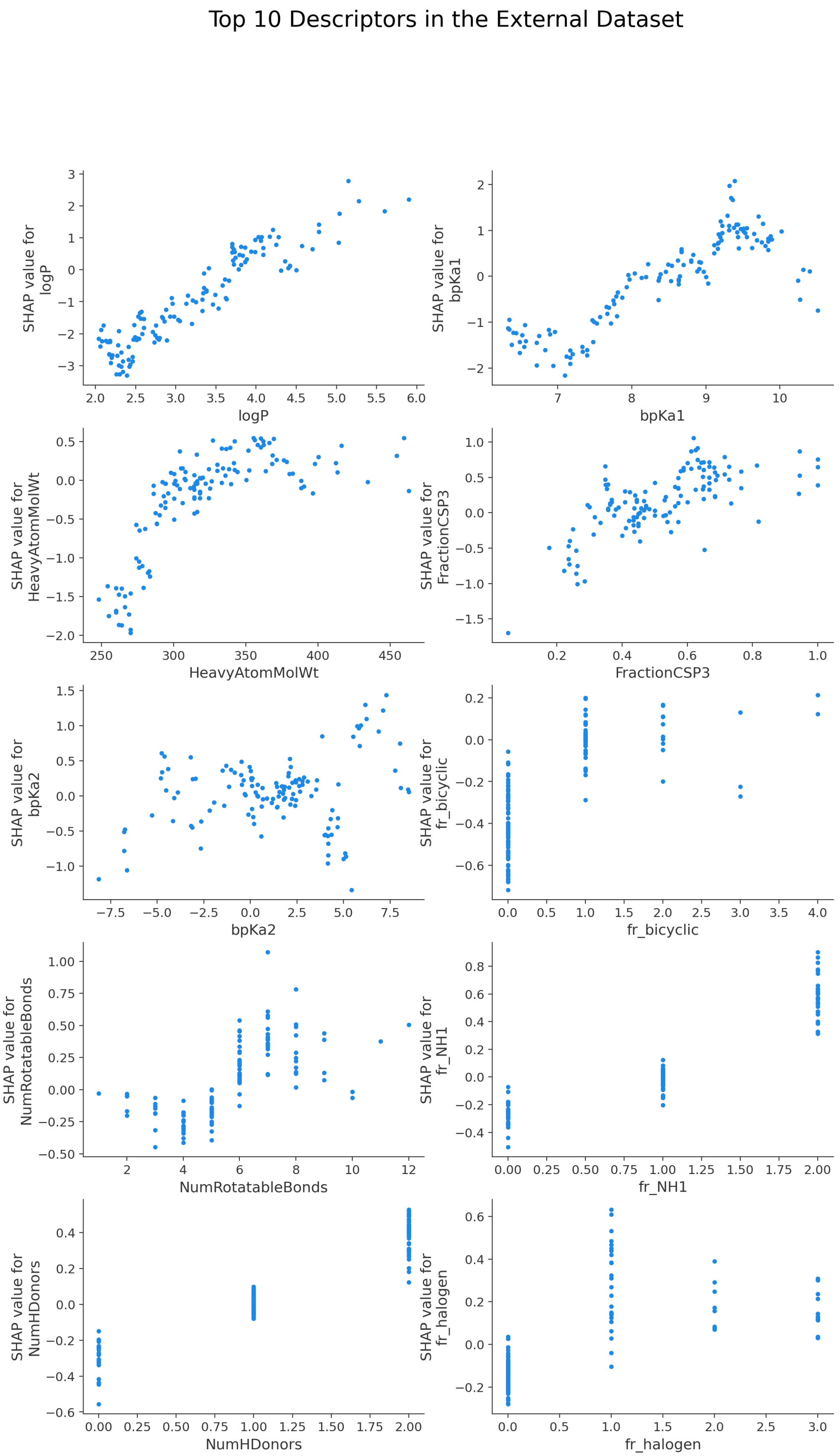

Figure S4: Descriptor model: Top 10 descriptors' Dependence Plots in the external dataset.

Figure S5: Fingerprint model: X-FP report for the Training Set

Training Data: X-FP Report

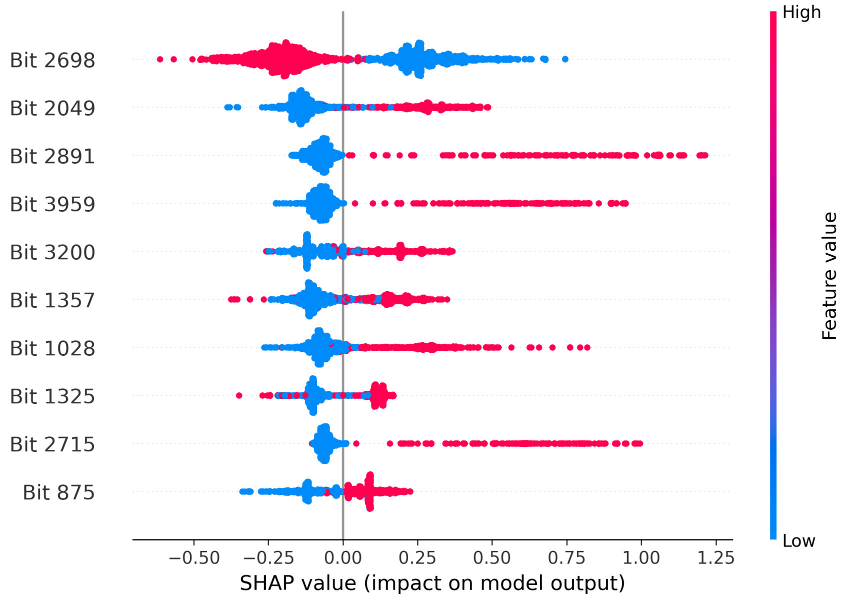

SHAP Summary Plot for the Top 10 Morgan Fingerprint Bits

X-FP

Bit Analysis

Bit 2698

SHAP Summary Plot for the Bit 2698:

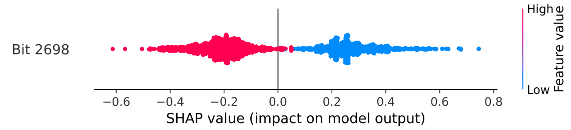

Substructures present:

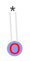

1

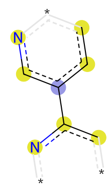

2

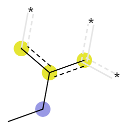

3

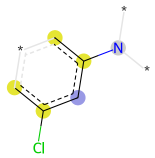

4

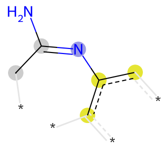

5

Substructures frequency table (total compounds: 2065):

Serial	Substructures (as SMARTS)	Total occurrences	Unique occurrences
1	[O]~*	6335	1743
2	[c;R]1(:[cH;R]:[cH;R]~*~[n;R]:[cH;R]:1)-[c;R](:[cH;R]~*):[n;R]~*	6	6
3	[CH2](-[CH3])-[c;R](:[cH;R]~*):[c;R](~*)~*	19	16
4	[cH;R]1:[c;R](:[cH;R]~*~[cH;R]:[c;R]:1-[N;R](~*)~*)-[Cl]	7	7
5	[N;R](=[C;R](-[NH2])-[CH2;R]~*)-[c;R](:[cH;R]~*):[c;R](~*)~*	1	1

Bit 2049

SHAP Summary Plot for the Bit 2049:

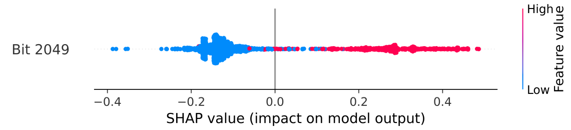

Substructures present:

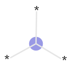

1

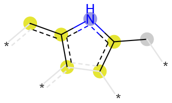

2

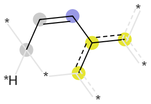

3

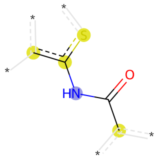

4

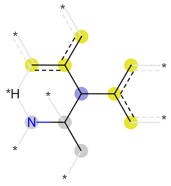

5

Substructures frequency table (total compounds: 2065):

Serial	Substructures (as SMARTS)	Total occurrences	Unique occurrences
1	[CH](~*)(~*)~*	3287	1463
2	[nH;R]1:[c;R](:[c;R](:[c;R]1:[cH;R]~*)~*)~*)-[CH2;R]~*	41	41
3	[CH;R]1-[c;R](:[c;R](~*)~*~[C;R](-[CH;R]=1)(~*)~*):[c;R](~*)~*	2	2
4	[NH](-[C](=[O])-[c;R](~*)~*)-[c;R](:[s;R]~*):[c;R](~*)~*	1	1
5	[CH;R]1(-[CH;R](-[N;R](~*)~*~[c;R](:[c;R]-1:[cH;R]~*)~*)-[CH;R](~*)~*)-[c;R](:[cH;R]~*):[cH;R]~*	3	3

X-FP

Bit Analysis

Bit 2891

SHAP Summary Plot for the Bit 2891:

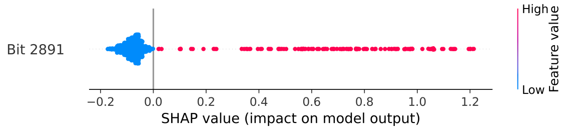

Substructures present:

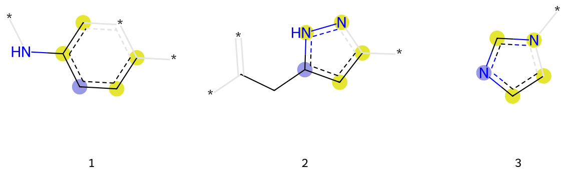

Substructures frequency table (total compounds: 2065):

Serial	Substructures (as SMARTS)	Total occurrences	Unique occurrences
1	[cH;R]1:[cH;R]:[c;R](~*)~*~[cH;R]:[c;R]:1-[NH]~*	221	170
2	[c;R]1(:[cH;R]:[c;R](:[n;R]:[nH;R]:1)~*)-[CH2]-[C](~*)~*	1	1
3	[n;R]1:[cH;R]:[cH;R]:[n;R](:[cH;R]:1)~*	12	11

X-FP

Bit Analysis

Bit 3959

SHAP Summary Plot for the Bit 3959:

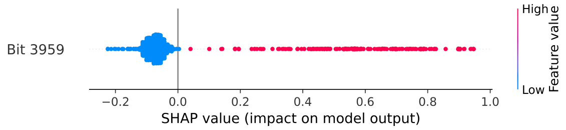

Substructures present:

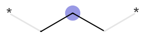

1

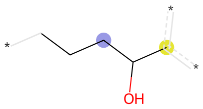

2

Substructures frequency table (total compounds: 2065):

Serial	Substructures (as SMARTS)	Total occurrences	Unique occurrences
1	[CH2](-[CH2]~*)-[CH2]~*	1251	650
2	[CH2](-[CH2]-[CH2]~*)-[CH](-[OH])-[c;R](~*)~*	2	2

X-FP

Bit Analysis

Bit 3200

SHAP Summary Plot for the Bit 3200:

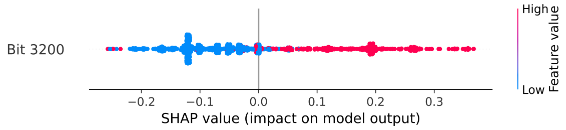

Substructures present:

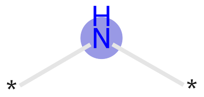

1

Substructures frequency table (total compounds: 2065):

Serial	Substructures (as SMARTS)	Total occurrences	Unique occurrences
1	[NH](~*)~*	1334	1033

Bit 1357

SHAP Summary Plot for the Bit 1357:

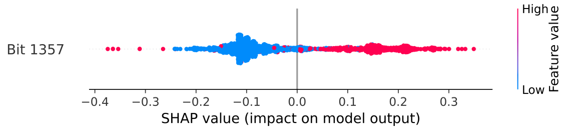

Substructures present:

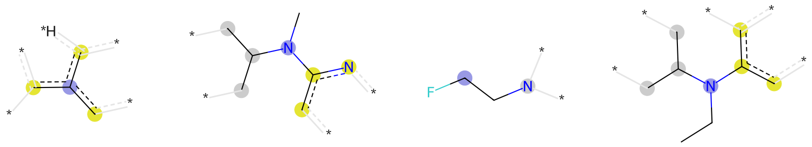

1 2 3 4

Substructures frequency table (total compounds: 2065):

Serial	Substructures (as SMARTS)	Total occurrences	Unique occurrences
1	[c;R](:[cH;R]~*)(:[c;R](~*)~*):[c;R](~*)~*	729	687
2	[N](-[CH3])(-[CH;R](-[CH2;R]~*)-[CH2;R]~*)-[c;R](:[cH;R]~*):[n;R]~*	5	5
3	[CH2](-[F])-[CH2]-[N;R](~*)~*	1	1
4	[N](-[CH2]-[CH3])(-[CH;R](-[CH2;R]~*)-[CH2;R]~*)-[c;R](:[cH;R]~*):[c;R](~*)~*	2	2

Bit 1028

SHAP Summary Plot for the Bit 1028:

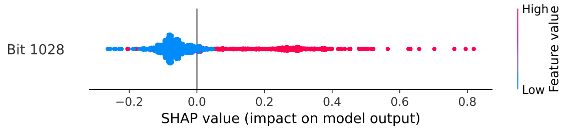

Substructures present:

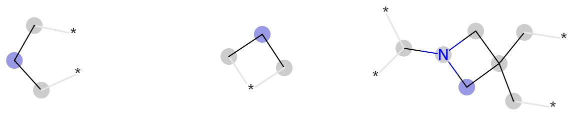

1 2 3

Substructures frequency table (total compounds: 2065):

Serial	Substructures (as SMARTS)	Total occurrences	Unique occurrences
1	[CH2;R](-[CH2;R]~*)-[CH2;R]~*	810	474
2	[CH2;R]1-[CH2;R]~*~[CH2;R]-1	13	13
3	[CH2;R]1-[N;R](-[CH2;R]-[C;R]-1(-[CH2;R]~*)-[CH2;R]~*)-[CH;R](~*)~*	1	1

X-FP

Bit Analysis

Bit 1325

SHAP Summary Plot for the Bit 1325:

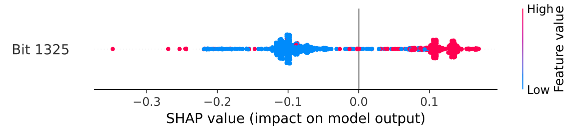

Substructures present:

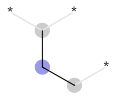

1

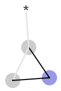

2

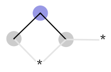

3

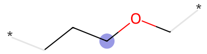

4

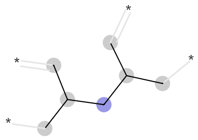

5

Substructures frequency table (total compounds: 2065):

Serial	Substructures (as SMARTS)	Total occurrences	Unique occurrences
1	[CH2;R](-[CH2;R]~*)-[CH;R](~*)~*	1093	779
2	[CH2;R]1-[CH2;R]-[CH;R]-1~*	23	22
3	[CH2;R]1-[CH2;R]~*~[CH;R]-1~*	6	6
4	[CH2](-[CH2]-[CH2]~*)-[O]-[CH2]~*	6	3
5	[CH2](-[CH;R](-[CH2;R]~*)-[CH;R]~*)-[CH;R](-[CH2;R]~*)-[CH;R]~*	1	1

X-FP

Bit Analysis

Bit 2715

SHAP Summary Plot for the Bit 2715:

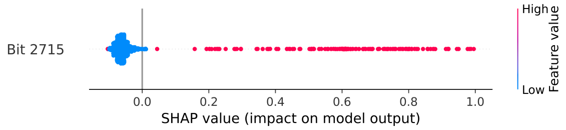

Substructures present:

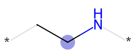

1

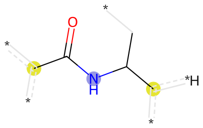

2

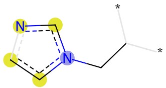

3

Substructures frequency table (total compounds: 2065):

Serial	Substructures (as SMARTS)	Total occurrences	Unique occurrences
1	[CH2](-[CH2]~*)-[NH]~*	349	304
2	[NH](-[CH](-[CH2]~*)-[c;R](~*)~*)-[C](=[O])-[c;R](~*)~*	1	1
3	[n;R]1(:[cH;R]:[cH;R]:[n;R]:[cH;R]:1)-[CH2]-[CH](~*)~*	6	6

Bit 875

SHAP Summary Plot for the Bit 875:

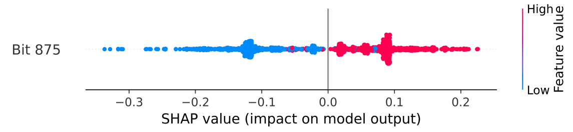

Substructures present:

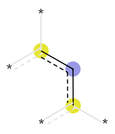
1

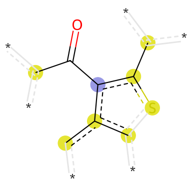
2

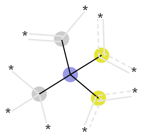
3

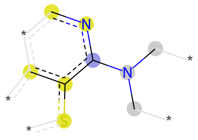
4

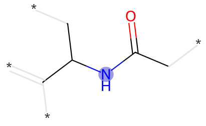
5

Substructures frequency table (total compounds: 2065):

Serial	Substructures (as SMARTS)	Total occurrences	Unique occurrences
1	[cH;R](:[c;R](~*)~*):[c;R](~*)~*	1809	1185
2	[c;R]1(:[c;R](:[s;R]:[c;R](:[c;R]:1:[cH;R]~*)~*)-[c;R](~*)~*)-[C](=[O])-[c;R](~*)~*	1	1
3	[C;R](-[C;R](~*)~*)(-[c;R](~*)~*)(-[c;R](~*)~*)-[C;R](~*)(~*)~*	27	27
4	[c;R]1(:[n;R]:[cH;R]~*~[c;R](:[c;R]:1:[s;R]~*)~*)-[N;R](-[CH2;R]~*)-[CH2;R]~*	2	2
5	[NH](-[CH](-[CH2]~*)-[C](~*)~*)-[C](=[O])-[CH2]~*	12	12

X-FP

Bit Analysis

Notes about substructures rendering:

- The molecule fragment is drawn with the atoms in the same positions as in the original molecule.
- The central atom is highlighted in blue.
- Aromatic atoms are highlighted in yellow.
- Aliphatic ring atoms are highlighted in dark grey.
- Atoms/bonds that are drawn in light grey indicate pieces of the structure that influence the atoms' connectivity invariants but that are not directly part of the fingerprint.

Notes about feature importance:

The feature importance here is done by SHAP TreeExplainer. Following are the notes on interpreting the beeswarm SHAP Summary Plots:

- Each dot is the SHAP value of a bit of a molecule (terms used interchangeably are: sample, observation, data point, entry, compound, etc.) in the dataset.
- The dots representing the same SHAP values of a bit overlap. However, the frequency of these values can be observed as the density of the dots along the x-axis.
- Morgan Fingerprint bits are binary in nature. A red dot shows that the bit is switched on for a molecule, meaning that at least one substructure present in this molecule is encoded by this bit. A blue dot means that the bit is switched off for a molecule and this means that no substructures are present in this molecule which are encoded by this bit.
- For binary classification, SHAP values of a Morgan Fingerprint bit greater than zero describe that the presence or absence of this feature contributes towards the model prediction classes' second category (usually labelled as '1'). While the SHAP values less than zero describe that the presence or absence of this feature contributes towards the first category (usually labelled as '0').

Caution:

- Bar plots will be used instead of beeswarm plots as a default SHAP Summary Plot in case of multi-class classification. X-FP analysis for multi-class classification is still under testing and should be completely avoided.
- X-FP analysis for regression models is also under testing, and while such models and their SHAP analysis are compatible, they should be also avoided for time-being.

Figure S6: Fingerprint model: X-FP report for the Time-Split Set

Time-Split Data: X-FP Report

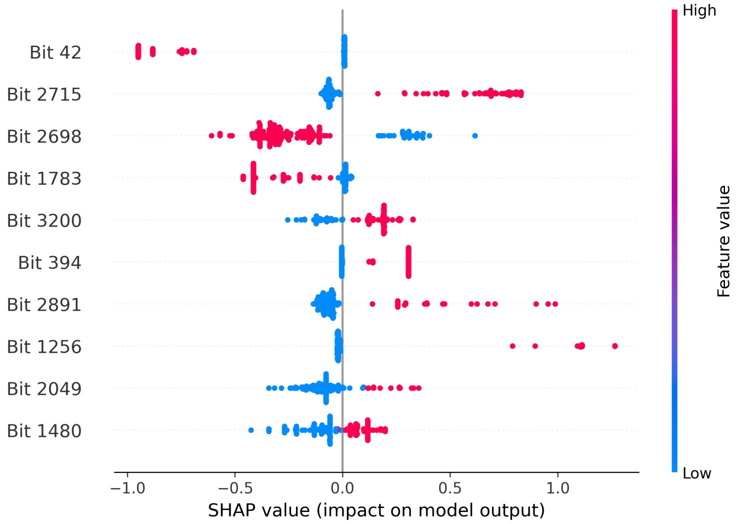

SHAP Summary Plot for the Top 10 Morgan Fingerprint Bits

X-FP

Bit Analysis

Bit 42

SHAP Summary Plot for the Bit 42:

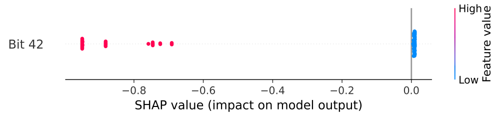

Substructures present:

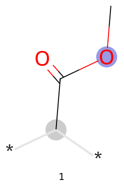

Substructures frequency table (total compounds: 156):

Serial	Substructures (as SMARTS)	Total occurrences	Unique occurrences
1	[O](-[CH3])-[C](=[O])-[CH;R](~*)~*	73	73

X-FP

Bit Analysis

Bit 2715

SHAP Summary Plot for the Bit 2715:

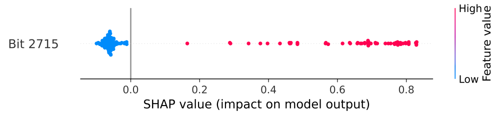

Substructures present:

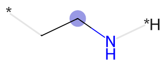

1

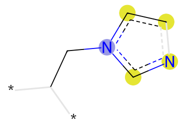

2

Substructures frequency table (total compounds: 156):

Serial	Substructures (as SMARTS)	Total occurrences	Unique occurrences
1	[CH2](-[CH2]~*)-[NH]~*	84	71
2	[n;R]1(:[cH;R]:[cH;R]:[n;R]:[cH;R]:1)-[CH2]-[CH](~*)~*	1	1

X-FP

Bit Analysis

Bit 2698

SHAP Summary Plot for the Bit 2698:

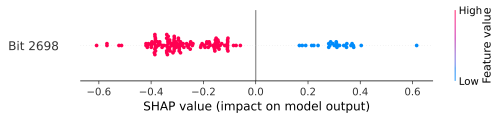

Substructures present:

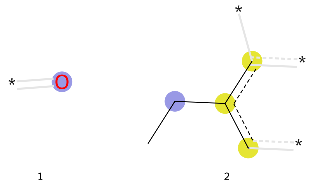

Substructures frequency table (total compounds: 156):

Serial	Substructures (as SMARTS)	Total occurrences	Unique occurrences
1	[O]~*	542	149
2	[CH2](-[CH3])-[c;R](:[cH;R]~*):[c;R](~*)~*	1	1

X-FP

Bit Analysis

Bit 1783

SHAP Summary Plot for the Bit 1783:

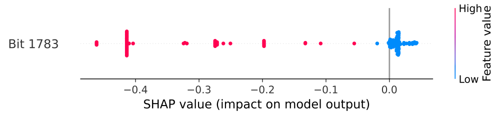

Substructures present:

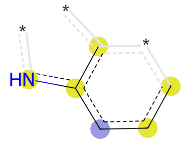

1

Substructures frequency table (total compounds: 156):

Serial	Substructures (as SMARTS)	Total occurrences	Unique occurrences
1	[cH;R]1:[cH;R]:[cH;R]~*~[c;R](:[c;R]:1:[nH;R]~*)~*	76	76

X-FP

Bit Analysis

Bit 3200

SHAP Summary Plot for the Bit 3200:

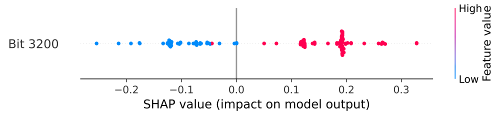

Substructures present:

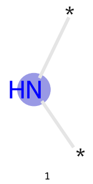

Substructures frequency table (total compounds: 156):

Serial	Substructures (as SMARTS)	Total occurrences	Unique occurrences
1	[NH](~*)~*	174	122

X-FP

Bit Analysis

Bit 394

SHAP Summary Plot for the Bit 394:

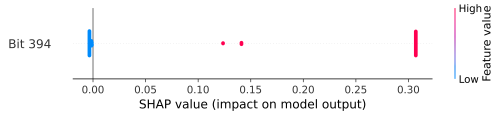

Substructures present:

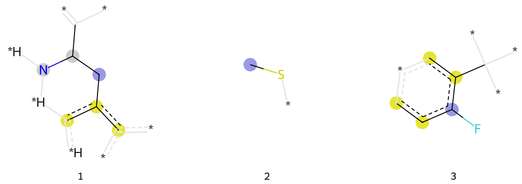

Substructures frequency table (total compounds: 156):

Serial	Substructures (as SMARTS)	Total occurrences	Unique occurrences
1	[CH2;R]1-[CH;R](-[N;R](~*)~*~[c;R](:[c;R]-1:[c;R](~*)~*)~*)-[C](~*)~*	73	73
2	[CH3]-[S]~*	2	2
3	[c;R]1(:[cH;R]:[cH;R]~*~[cH;R]:[c;R]:1-[C](~*)(~*)~*)-[F]	1	1

Bit 2891

SHAP Summary Plot for the Bit 2891:

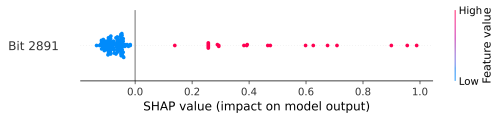

Substructures present:

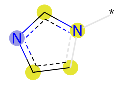

1

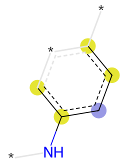

2

Substructures frequency table (total compounds: 156):

Serial	Substructures (as SMARTS)	Total occurrences	Unique occurrences
1	[n;R]1:[cH;R]:[cH;R]:[n;R](:[cH;R]:1)~*	11	11
2	[cH;R]1:[cH;R]:[c;R](~*)~*~[cH;R]:[c;R]:1-[NH]~*	12	12

X-FP

Bit Analysis

Bit 1256

SHAP Summary Plot for the Bit 1256:

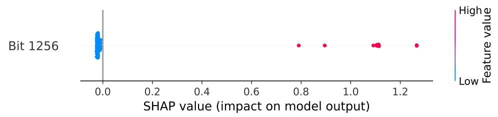

Substructures present:

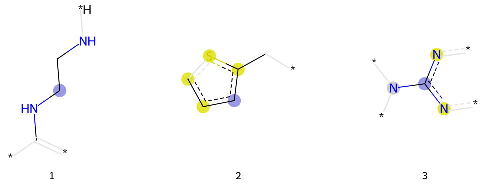

Substructures frequency table (total compounds: 156):

Serial	Substructures (as SMARTS)	Total occurrences	Unique occurrences
1	[CH2](-[CH2]-[NH]~*)-[NH]-[C](~*)~*	20	10
2	[cH;R]1:[cH;R]:[cH;R]:[s;R]:[c;R]:1-[CH2]~*	3	3
3	[c;R](:[n;R]~*)(:[n;R]~*)-[N;R](~*)~*	3	2

X-FP

Bit Analysis

Bit 2049

SHAP Summary Plot for the Bit 2049:

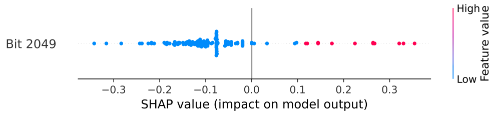

Substructures present:

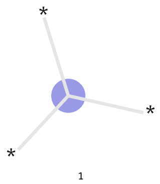

Substructures frequency table (total compounds: 156):

Serial	Substructures (as SMARTS)	Total occurrences	Unique occurrences
1	[CH](~*)(~*)~*	430	127

X-FP

Bit Analysis

Bit 1480

SHAP Summary Plot for the Bit 1480:

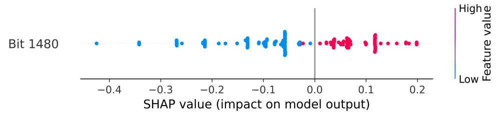

Substructures present:

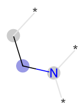

1

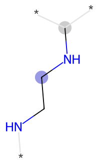

2

Substructures frequency table (total compounds: 156):

Serial	Substructures (as SMARTS)	Total occurrences	Unique occurrences
1	[CH2;R](-[CH2;R]~*)-[N;R](~*)~*	134	61
2	[CH2](-[CH2]-[NH]~*)-[NH]-[CH;R](~*)~*	10	10

X-FP

Bit Analysis

Notes about substructures rendering:

- The molecule fragment is drawn with the atoms in the same positions as in the original molecule.
- The central atom is highlighted in blue.
- Aromatic atoms are highlighted in yellow.
- Aliphatic ring atoms are highlighted in dark grey.
- Atoms/bonds that are drawn in light grey indicate pieces of the structure that influence the atoms' connectivity invariants but that are not directly part of the fingerprint.

Notes about feature importance:

The feature importance here is done by SHAP TreeExplainer. Following are the notes on interpreting the beeswarm SHAP Summary Plots:

- Each dot is the SHAP value of a bit of a molecule (terms used interchangeably are: sample, observation, data point, entry, compound, etc.) in the dataset.
- The dots representing the same SHAP values of a bit overlap. However, the frequency of these values can be observed as the density of the dots along the x-axis.
- Morgan Fingerprint bits are binary in nature. A red dot shows that the bit is switched on for a molecule, meaning that at least one substructure present in this molecule is encoded by this bit. A blue dot means that the bit is switched off for a molecule and this means that no substructures are present in this molecule which are encoded by this bit.
- For binary classification, SHAP values of a Morgan Fingerprint bit greater than zero describe that the presence or absence of this feature contributes towards the model prediction classes' second category (usually labelled as '1'). While the SHAP values less than zero describe that the presence or absence of this feature contributes towards the first category (usually labelled as '0').

Caution:

- Bar plots will be used instead of beeswarm plots as a default SHAP Summary Plot in case of multi-class classification. X-FP analysis for multi-class classification is still under testing and should be completely avoided.
- X-FP analysis for regression models is also under testing, and while such models and their SHAP analysis are compatible, they should be also avoided for time-being.

Figure S7: Fingerprint model: X-FP report for the External Set

Purchase Data: X-FP Report

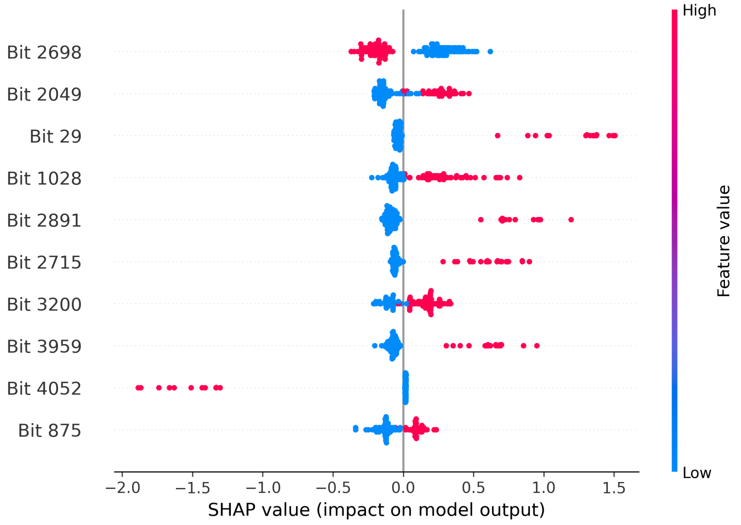

SHAP Summary Plot for the Top 10 Morgan Fingerprint Bits

Purchase Data: X-FP Report *Page 1 of 12* *Report generated on 2023-10-12 20:57:25*

X-FP

Bit Analysis

Bit 2698

SHAP Summary Plot for the Bit 2698:

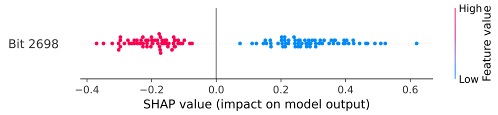

Substructures present:

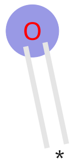

1

Substructures frequency table (total compounds: 127):

Serial	Substructures (as SMARTS)	Total occurrences	Unique occurrences
1	[O]~*	236	95

X-FP

Bit Analysis

Bit 2049

SHAP Summary Plot for the Bit 2049:

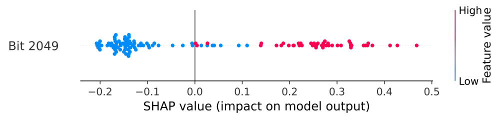

Substructures present:

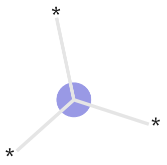

1

Substructures frequency table (total compounds: 127):

Serial	Substructures (as SMARTS)	Total occurrences	Unique occurrences
1	[CH](~*)(~*)~*	157	87

Bit 29

SHAP Summary Plot for the Bit 29:

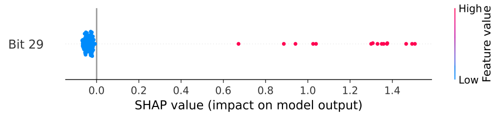

Substructures present:

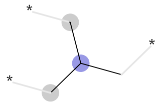

1

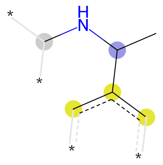

2

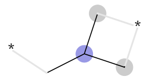

3

Substructures frequency table (total compounds: 127):

Serial	Substructures (as SMARTS)	Total occurrences	Unique occurrences
1	[CH;R](-[CH2;R]~*)(-[CH2;R]~*)-[CH2]~*	20	15
2	[CH](-[CH3])(-[NH]-[CH;R](~*)~*)-[c;R](:[cH;R]~*):[cH;R]~*	1	1
3	[CH;R]1(-[CH2;R]~*~[CH2;R]-1)-[CH2]~*	1	1

X-FP

Bit Analysis

Bit 1028

SHAP Summary Plot for the Bit 1028:

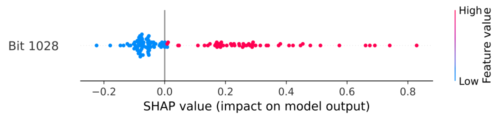

Substructures present:

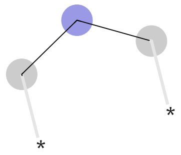

1

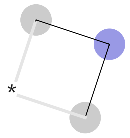

2

Substructures frequency table (total compounds: 127):

Serial	Substructures (as SMARTS)	Total occurrences	Unique occurrences
1	[CH2;R](-[CH2;R]~*)-[CH2;R]~*	94	53
2	[CH2;R]1-[CH2;R]~*~[CH2;R]-1	1	1

X-FP

Bit Analysis

Bit 2891

SHAP Summary Plot for the Bit 2891:

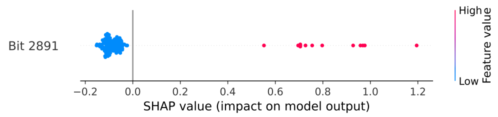

Substructures present:

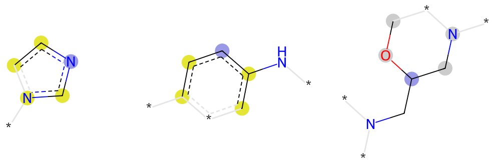

Substructures frequency table (total compounds: 127):

Serial	Substructures (as SMARTS)	Total occurrences	Unique occurrences
1	[n;R]1:[cH;R]:[cH;R]:[n;R](:[cH;R]:1)~*	2	2
2	[cH;R]1:[cH;R]:[c;R](~*)~*~[cH;R]:[c;R]:1-[NH]~*	10	10
3	[CH;R]1(-[CH2;R]-[N;R](~*)~*~[CH2;R]-[O;R]-1)-[CH2]-[N](~*)~*	1	1

X-FP

Bit Analysis

Bit 2715

SHAP Summary Plot for the Bit 2715:

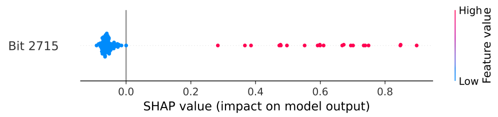

Substructures present:

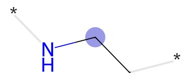

1

Substructures frequency table (total compounds: 127):

Serial	Substructures (as SMARTS)	Total occurrences	Unique occurrences
1	[CH2](-[CH2]~*)-[NH]~*	28	26

Bit 3200

SHAP Summary Plot for the Bit 3200:

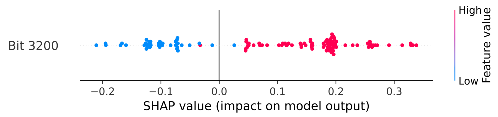

Substructures present:

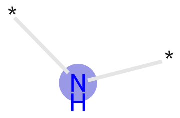

1

Substructures frequency table (total compounds: 127):

Serial	Substructures (as SMARTS)	Total occurrences	Unique occurrences
1	[NH](~*)~*	108	91

X-FP

Bit Analysis

Bit 3959

SHAP Summary Plot for the Bit 3959:

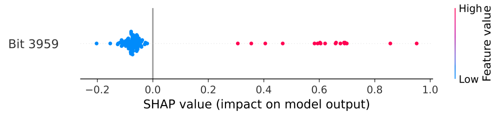

Substructures present:

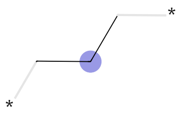

1

Substructures frequency table (total compounds: 127):

Serial	Substructures (as SMARTS)	Total occurrences	Unique occurrences
1	[CH2](-[CH2]~*)-[CH2]~*	115	64

X-FP

Bit Analysis

Bit 4052

SHAP Summary Plot for the Bit 4052:

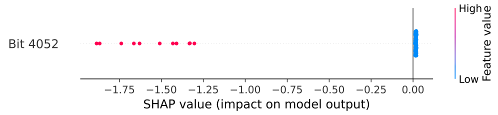

Substructures present:

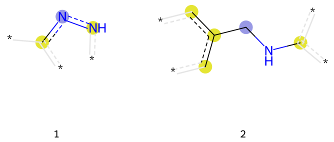

Substructures frequency table (total compounds: 127):

Serial	Substructures (as SMARTS)	Total occurrences	Unique occurrences
1	[n;R](:[nH;R]~*):[c;R](~*)~*	9	9
2	[CH2](-[NH]-[c;R](~*)~*)-[c;R](:[cH;R]~*):[cH;R]~*	2	2

X-FP

Bit Analysis

Bit 875

SHAP Summary Plot for the Bit 875:

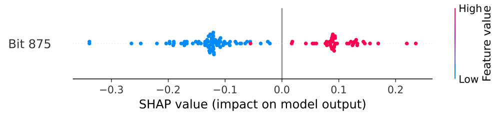

Substructures present:

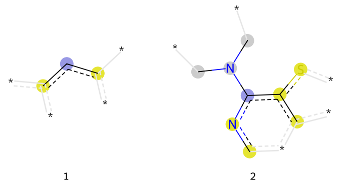

Substructures frequency table (total compounds: 127):

Serial	Substructures (as SMARTS)	Total occurrences	Unique occurrences
1	[cH;R](:[c;R](~*)~*):[c;R](~*)~*	56	46
2	[c;R]1(:[n;R]:[cH;R]~*~[c;R](:[c;R]:1:[s;R]~*)~*)-[N;R](-[CH2;R]~*)-[CH2;R]~*	1	1

X-FP

Bit Analysis

Notes about substructures rendering:

- The molecule fragment is drawn with the atoms in the same positions as in the original molecule.
- The central atom is highlighted in blue.
- Aromatic atoms are highlighted in yellow.
- Aliphatic ring atoms are highlighted in dark grey.
- Atoms/bonds that are drawn in light grey indicate pieces of the structure that influence the atoms' connectivity invariants but that are not directly part of the fingerprint.

Notes about feature importance:

The feature importance here is done by SHAP TreeExplainer. Following are the notes on interpreting the beeswarm SHAP Summary Plots:

- Each dot is the SHAP value of a bit of a molecule (terms used interchangeably are: sample, observation, data point, entry, compound, etc.) in the dataset.
- The dots representing the same SHAP values of a bit overlap. However, the frequency of these values can be observed as the density of the dots along the x-axis.
- Morgan Fingerprint bits are binary in nature. A red dot shows that the bit is switched on for a molecule, meaning that at least one substructure present in this molecule is encoded by this bit. A blue dot means that the bit is switched off for a molecule and this means that no substructures are present in this molecule which are encoded by this bit.
- For binary classification, SHAP values of a Morgan Fingerprint bit greater than zero describe that the presence or absence of this feature contributes towards the model prediction classes' second category (usually labelled as '1'). While the SHAP values less than zero describe that the presence or absence of this feature contributes towards the first category (usually labelled as '0').

Caution:

- Bar plots will be used instead of beeswarm plots as a default SHAP Summary Plot in case of multi-class classification. X-FP analysis for multi-class classification is still under testing and should be completely avoided.
- X-FP analysis for regression models is also under testing, and while such models and their SHAP analysis are compatible, they should be also avoided for time-being.

Table S1: List of selected intuitive RDKit descriptors (version 2022.09.05)

1	NumValenceElectrons
2	NumRadicalElectrons
3	NumAliphaticCarbocycles
4	NumAliphaticHeterocycles
5	NumAliphaticRings
6	NumAromaticCarbocycles
7	NumAromaticHeterocycles
8	NumAromaticRings
9	NumHAcceptors
10	NumHDonors
11	NumHeteroatoms
12	NumRotatableBonds
13	NumSaturatedCarbocycles
14	NumSaturatedHeterocycles
15	NumSaturatedRings
16	HeavyAtomMolWt
17	TPSA
18	FractionCSP3
19	HeavyAtomCount
20	NHOHCount
21	NOCount
22	RingCount
23	fr_Al_COO
24	fr_Al_OH
25	fr_Al_OH_noTert
26	fr_ArN
27	fr_Ar_COO
28	fr_Ar_N
29	fr_Ar_NH
30	fr_Ar_OH
31	fr_COO
32	fr_COO2
33	fr_C_O
34	fr_C_O_noCOO
35	fr_C_S
36	fr_HOCCN
37	fr_Imine
38	fr_NH0
39	fr_NH1
40	fr_NH2
41	fr_N_O
42	fr_Ndealkylation1
43	fr_Ndealkylation2
44	fr_Nhpyrrole
45	fr_SH
46	fr_aldehyde
47	fr_alkyl_carbamate
48	fr_alkyl_halide
49	fr_allylic_oxid
50	fr_amide
51	fr_amidine
52	fr_aniline
53	fr_aryl_methyl
54	fr_azide
55	fr_azo
56	fr_barbitur

57 fr_benzene
58 fr_benzodiazepine
59 fr_bicyclic
60 fr_diazo
61 fr_dihydropyridine
62 fr_epoxide
63 fr_ester
64 fr_ether
65 fr_furan
66 fr_guanido
67 fr_halogen
68 fr_hdrzine
69 fr_hdrzone
70 fr_imidazole
71 fr_imide
72 fr_isocyan
73 fr_isothiocyan
74 fr_ketone
75 fr_ketone_Topliss
76 fr_lactam
77 fr_lactone
78 fr_methoxy
79 fr_morpholine
80 fr_nitrile
81 fr_nitro
82 fr_nitro_arom
83 fr_nitro_arom_nonortho
84 fr_nitroso
85 fr_oxazole
86 fr_oxime
87 fr_para_hydroxylation
88 fr_phenol
89 fr_phenol_noOrthoHbond
90 fr_phos_acid
91 fr_phos_ester
92 fr_piperdine
93 fr_piperzine
94 fr_priamide
95 fr_prisulfonamd
96 fr_pyridine
97 fr_quatN
98 fr_sulfide
99 fr_sulfonamd
100 fr_sulfone
101 fr_term_acetylene
102 fr_tetrazole
103 fr_thiazole
104 fr_thiocyan
105 fr_thiophene
106 fr_unbrch_alkane
107 fr_urea
